# Molecular speciation controls arsenic and lead bioaccessibility in fugitive dusts from sulfidic mine tailings†

**DOI:** 10.1039/d2em00182a

**Published:** 2022-10-03

**Authors:** Robert A. Root, Jon Chorover

**Affiliations:** a Department of Environmental Science, University of Arizona Tucson AZ USA rroot@arizona.edu +1520-626-6782 +1520-626-1709; b Arizona Laboratory for Emerging Contaminants, University of Arizona Tucson AZ USA

## Abstract

Communities nearby mine wastes in arid and semi-arid regions are potentially exposed to high concentrations of toxic metal(loid)s from fugitive dusts deriving from impoundments. To assess the relation between potentially lofted particles and human health risk, we studied the relationship between pharmacokinetic bioaccessibility and metal(loid) molecular speciation for mine tailings dust particulate matter (PM), with elevated levels of arsenic and lead (up to 59 and 34 mmol kg^−1^, respectively), by coupling *in vitro* bioassay (IVBA) with X-ray absorption spectroscopy (XAS). Mine tailing efflorescent salts (PM_ES_) and PM from the surface crust (0–1 cm, PM_SC_) and near surface (0–25 cm) were isolated to <10 μm and <150 μm effective spherical diameter (PM_10_ and PM_150_) and reacted with synthetic gastric and lung fluid for 30 s to 100 h to investigate toxic metal(loid) release kinetics. Bioaccessible (BAc) fractions of arsenic and lead were about 10 and 100 times greater in gastric than in lung fluid simulant, respectively, and 10–100% of the maximum gastric BAc from PM_10_ and PM_150_ occurred within 30 s, with parabolic dissolution of fine, highly-reactive particles followed by slower release from less soluble sources. Evaporite salts were almost completely solubilized in gastric-fluid simulants. Arsenate within jarosite and sorbed to ferrihydrite, and lead from anglesite, were identified by XAS as the principal contaminant sources in the near surface tailings. In the synthetic lung fluid, arsenic was released continuously to 100 h, suggesting that residence time *in vivo* must be considered for risk determination. Analysis of pre- and post-IVBA PM indicated the release of arsenic in lung fluid was principally from arsenic-substituted jarosite, whereas in synthetic gastric fluid arsenic complexed on ferrihydrite surfaces was preferentially released and subsequently repartitioned to jarosite-like coordination at extended exposures. Lead dissolved at 30 s was subsequently repartitioned back to the solid phase as pyromorphite in phosphate rich lung fluid. The bioaccessibility of lead in surface tailings PM was limited due to robust sequestration in plumbojarosite. Kinetic release of toxic elements in both synthetic biofluids indicated that a single IVBA interval may not adequately describe release dynamics.

## Abstract 2

Mine tailings are sources of metal(loid)-rich particulate matter (PM), particularly in dry climates. Inhaled or ingested PM bioavailability and toxicity depends on the solid phase speciation and the rate of PM dissolution.

## Graphical abstract

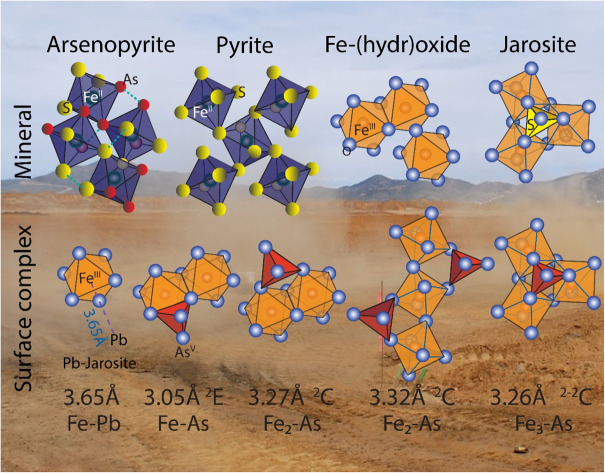

**Environmental significance**

Human health risks from particulate matter (PM) are becoming increasingly clear. Mine tailings serve as PM sources, especially in dry climates. The US Agency for Toxic Substances and Disease Registry shows the top 2 substances of concern are arsenic (#1) and lead (#2), metal(loid)s commonly found in mine tailings. With the associated likelihood of off-site dust transport, inhalation and ingestion of PM can lead to toxic exposures with biological effects controlled by the rate of dissolution *in vivo*. However, the bioavailability, toxicity, and uptake of metal(loid)s also depends on their solid phase speciation, which can be probed with X-ray spectroscopy. This study uniquely combines pharmacokinetic bioassay and solid phase speciation to determine toxic metal(loid) source and kinetic release *in vitro*.

## 1 Introduction

Mine tailings are a significant source of fugitive dust pollution in the form of atmospheric particulate matter (PM). Since fine-grained, unconsolidated, metalliferous particles can be subjected to suspension and transport by wind (and water), potentially resulting in direct human exposure to toxicants, there is a need to know the lability and precise molecular form or speciation of metal(loid)s that may emit from a tailings weathering environment.^1^ Previous studies point to aeolian transport of fugitive dusts as a primary dispersal mechanism for tailings particles.^2^ As such, there is a human health risk of exposure from ingestion and inhalation of fugitive aerosols from mine tailings.^3–5^ However, the chemistry and bioavailability of arsenic and lead in dusts from mine wastes are less studied,^6–9^ particularly for wastes undergoing diagenetic alteration in (semi)-arid environments.^10–15^ Because tailing impoundments often remain barren for decades to centuries due to conditions inhospitable to plant and microbial growth, they are particularly susceptible to erosive forces of wind and rain.^16,17^ Exposure to airborne geodusts is expected to increase as climate change intensifies the aridity of drylands.^18^ Direct human exposure to PM results in toxic exposures with biological effects that are controlled by the pharmacokinetic rates of PM dissolution in biofluids.^2,19,20^

Primary routes of exposure to geo-dust toxicants include ingestion, inhalation, and dermal absorption. Whereas dermal absorption has been shown to be insignificant for arsenic and lead exposure in humans, fine particles adhered to the skin may be ingested by hand-to-mouth activities, which can be especially problematic for children in proximity to mine tailings.^21^ Inhalable and ingestible particles, generally <150 μm effective spherical diameter (ESD), can enter the mouth and nose, whereas particles < 10 μm ESD can pass into the lung. Larger particles that do not pass into the lung (>10 μm ESD) are trapped by cilia as part of the body's filtration defense and are subsequently swallowed.^22^ This defensive mechanism protects against inert dust particles that are ingested and excreted, but soluble toxic particles pose an exposure risk.^4^ Therefore, both gastric and lung biofluids are relevant considerations for inhalable PM.

Airborne dusts are of particular concern because they are well-known to be associated with widespread lung diseases such as pneumoconiosis (black lung and silicosis) and coccidioidomycosis (Valley Fever). When arsenic and lead are present in tailings-derived dust, toxicity effects present significant human health risk even at low mass concentrations.^23^ Exposure to arsenic has negative effects on nearly all body systems, acting as a multi-organ carcinogen, and it has been linked to type-2 diabetes.^24–27^ Whereas lead exposure results in toxic poisoning, especially at higher levels of inhalation and ingestion exposure,^28^ the principal concern with lead is systemic pediatric neurotoxicity,^29^ because studies have shown that young children, with greater cell development compared to adults, experience greater deleterious health impacts to environmental exposures.^30^

Assessment of risk to health from ingested or inhaled contaminants must account for the total toxicant concentration (*C*_i_) and its bioactivity (*a*_i-bio_), the fraction absorbed at a target organ.^31^ Animal model *in vivo* assays have been used to monitor contaminant uptake and elimination to access relative bioavailability (RBa), and *in vitro* bioassays (IVBA) have been used to determine solubilization of particles under lab-controlled biomimicry to access bioaccessibility (BAc), conditionally-defined as the concentration of an analyte of interest (*e.g.*, As, Pb) released to solution in biofluid simulants.^32,33^ Necessarily, total *C*_i_ ≥ BAc ≥ RBa ≥ *a*_ibio_, making BAc a conservative proxy for *a*_i-bio_. Whereas prior studies have used IVBA methods to assess arsenic^34,35^ and lead^20,36^ release from mine tailings, there are few studies that have employed concurrent molecular speciation analysis to test for relations of BAc and local coordination environment of contaminants.^37–39^

We hypothesized that arsenic and lead BAc values would be correlated with their molecular speciation for a range of samples derived from a mine tailings Superfund Site. Therefore, in the present work, arsenic and lead containing sulfide-ore tailings, which had undergone pedogenic weathering in a semi-arid climate, were subjected to IVBA and the time-course of reaction was interrogated by molecular spectroscopy to determine molecular controls over BAc of metalliferous wastes.^15,40^ Specifically, we combined macroscopic studies of particle dissolution kinetics in synthetic lung and gastric biofluids with synchrotron X-ray absorption spectroscopy (XAS) of contaminant arsenic and lead, and mineral host sulfur and iron speciation before, during, and after biofluid exposures. Synchrotron XAS proved to be a powerful tool for determining molecular controls over rates of arsenic and lead release to biofluids. The concurrent assessment of BAc and its molecular-level controls can improve risk assessments and inform approaches to remediation action.

## 2 Materials and methods

### 2.1 Sampling and characterization

Particulate samples, designated as geo-dust toxic metal sources, were isolated from the surface of tailings impoundments at the Iron King Mine Humboldt Smelter Superfund Site in Dewey-Humboldt, Arizona (see Fig. S1†). Four sample types were collected: (i) PM < 10 μm ESD (PM_10_) and (ii) PM < 150 μm ESD (PM_150_), both from the bulk surface (0–25 cm depth) tailings; (iii) surface crust (PM_SC_) collected at the air-tailings interface from the top 0–1 cm (sieved to < 150 μm ESD); and (iv) efflorescent salts (PM_ES_), representing those particles that precipitate as ponded mine waters evaporate, collected from dehydration of irrigated tailings used in a greenhouse experiment.^41^ These fine-grained particles represent those emitting as fugitive dust from tailings that could be ingested or inhaled.^2,4,23,42,43^ Each fraction was air dried at RT and sieved to obtain PM_150_. Isolation of the PM_10_ was performed *via* cyclone separation, which allows collection of sufficient mass for replicated IVBA reactors without any mechanical crushing, for details see.^44,45^

Total elemental concentrations were analyzed by inductively coupled plasma mass spectrometry (ICP-MS, PerkinElmer, Elan DRC-II) following microwave assisted dissolution by aqua regia (Arizona Laboratory for Emerging Contaminants, ALEC, University of Arizona) and HF, HNO_3_, HClO_4_, and HCl (Activation Labs, Ontario CA). All chemicals were reagent grade or better and all labware was acid washed and metal-free. Total arsenic was analyzed by instrumental neutron activation analysis (INAA) and total iron was analyzed by ICP-AES following dissolution of the tailings in a lithium metaborate flux-fusion (with LiBO_2_ and Li_2_B_4_O_7_) (Activation Labs, Ontario CA). Certified reference materials were digested and analyzed along with the tailing samples with an acceptance range of ± 10% of the certified value to verify precision and accuracy in sample preparation and analysis. Specific surface area (SSA) was measured by multipoint BET (Brunauer, Emmett, and Teller, Micromeritics Gemini VII 2390 t, Norcross, GA).

### 2.2 Bioaccessibility of metal(loid)s

BAc was used to assess the metal(loid) exposure risk by monitoring the concentration of As, Pb, and host mineral Fe solubilized into IVBA synthetic biofluid and available for biologic absorption.^8,45,46^1
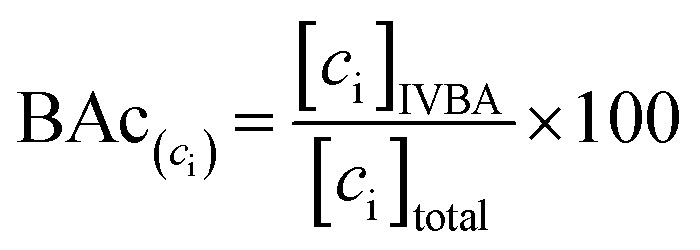
where the BAc fraction of element *c*_i_ is the concentration dissolved (mmol kg^−1^) in synthetic lung fluid (SLF) or synthetic gastric fluid (SGF) [(*c*_i_)_IVBA_] normalized to the total solid phase concentration [(*c*_i_)_total_] in the tailings PM sample.

The lung fluid simulant was a modified Gamble's solution, used previously to mimic the fluid released by Type II alveolar cells and estimate the potential *in vivo* dissolution of metal(loids) following inhalation of dusts.^47–49^ The solution contained a mixture of mineral salts, humectants, and an emulsifier, but omitted proteins to eschew putrefaction (Table S1†), and has been found to be analogous to human alveolar fluid in terms of major components.^50,51^ This SLF recipe has been used to determine the BAc of various respirable soil particles including, silica,^52^ cadmium,^53^ uranium,^54^ and lead.^55^

It has been established that among the many *in vitro* gastric extractions, a robust predictor of *in vivo* bioavailability, *i.e.*, a consistently good correlation between *in vitro* BAc and *in vivo* RBa with juvenile swine model for gastric exposures, is a low pH (1.5) fluid buffered with hydrochloric acid and glycine.^56,57^ Whereas the large swine body size is favorable for repeated blood sampling, this requires surgery, and swine are expensive and difficult to handle in a laboratory setting.^58^ For comparing *in vitro* and *in vivo* bioassays, mouse models are favorable due to their smaller size, lower expense, availability, relative ease of use and are already widely applied in medical research.^1,59–62^ However, mouse arsenic metabolism differs from that of humans and their small body weight and blood volume is prohibitive for repeat blood sampling.^63,64^ Despite these complications, mice and swine are both considered suitable animal bioassays for validation of *in vitro* bioavailability studies. The chosen IVBA method in this study is representative of both USEPA Method 1340 and the Solubility Bioaccessibility Research Consortium (SRC) assay.^39,65^ This SGF mimics conditions of a fasting stomach and was adopted by USEPA bioavailability protocol.^66,67^ The SGF, prepared in N_2_ sparged deionized water (Milli-Q, 18.2 MΩ) adjusted to pH 1.5 with concentrated HCl (ACS grade) and buffered with 0.4 M glycine, has been established to be appropriate for mining wastes^68^ and mine impacted soils, and has been validated as a good predictor of arsenic RBa in contaminated soil.^1,57^ This single-step gastric fluid approach was used in the current study.^66,69–71^

Kinetic IVBA experiments with SLF and SGF were carried out in triplicate in batch-mode. In each IVBA vessel, a 0.15 g aliquot of tailings PM was reacted with 15.0 g of SLF or SGF in a metal-free polypropylene tube (VWR, PN 89049-172). The IVBA solution was pre-heated to 37.0 °C to simulate internal body temperature and added to the tailings. The vessel was wrapped with aluminum foil to eschew photoreaction, placed on an end over end rotator in a 37.0 °C incubator at 60 rpm for prescribed durations. Reaction times <5 min were agitated by hand in pre-heated (37.0 °C) biofluid. At each interval, samples were removed from the rotator, centrifuged at 23 600 g, and the supernatant solution was aspirated and syringe filtered to 0.45 μm with acid washed hydrophobic polypropylene GHP membranes (acro-disc, Pall Corp. PN 28139). The filtered supernatant was analyzed for pH to assure changes were <0.5 and by ICP-MS for the elemental BAc concentrations. The concentration of the target analyte that migrated from the solid to aqueous phase was operationally defined as the kinetic-step BAc concentration. The residue was washed with DI water and lyophilized at −45 °C and 0.013 kPa prior to solid phase characterization and spectroscopic analysis. Kinetic studies of the tailing samples included five sample times for the PM_ES_ and PM_SC_ from 0.5 h to 48 h, and nine steps from 30 s to 100 h for the PM_10_ and PM_150_ tailings. Exposure durations were selected to develop (i) relevant particle resident times in lung (days) and gastric (hours) systems and (ii) an understanding of the kinetic release rates of metal(loid)s in these systems at shorter and longer retention times.

### 2.3 X-ray techniques

Bulk PM minerals were identified on pre- and post-IVBA reacted tailings using synchrotron transmission X-ray diffraction (ST-XRD) at 0.965 Å with a CCD detector for collection of Laue images at beamline 11–3 at the Stanford Synchrotron Radiation Lightsource (SSRL, Menlo Park, CA). Diffractograms were converted to conventional Cu Kα wavelength and normalized to the *hkl*_112_ quartz reflection (1.818 Å) for comparative analysis. Synchrotron X-ray absorption near-edge spectroscopy (XANES) and extended X-ray absorption fine structure (EXAFS) spectra were collected for speciation of arsenic, iron, (K-edge, 11 867 and 7112 eV) and lead (L_III_-edge 13 035 eV) at beamline 11–2 at SSRL. Sulfur (K-edge 2472.04 eV) XANES were collected at beamline 4–3 at SSRL. General procedures for XAS collection and data processing were described previously.^15^ The mixed ferric phases in PM were resolved using a non-linear least-squares fit to the Fe EXAFS constrained by a two-mineral model for shell-by-shell fits, simultaneously fitting a jarosite model component and a ferrihydrite model component to the tailing PM samples. Details of sample handling, preparation, data collection and analysis are provided in the (ESI†).

## 3 Results

### 3.1 Characterization of particulate matter

Both arsenic and lead in the tailing samples exceed the regional background levels ([As]_bkg_ = 0.33 mmol kg^−1^, [Pb]_bkg_ = 0.10 mmol kg^−1^),^72^ with tailing PM exhibiting multi-metal enrichment.^15^ Total metal(loid) concentration increased with decreasing particle-size, consistent with previous studies of mine tailings^8,12^ (Table 1). The iron-normalized molar concentrations of arsenic and lead (As : Fe, Pb : Fe) varied by sample type, with values of about 0.02 and 0.01, respectively, for PM_10_ and PM_SC_, while PM_150_ had lower values of about 0.015 and 0.005, respectively, indicating a relative enrichment in both arsenic and lead in PM_10_. The PM_ES_, which had <63 μm ESD, were completely soluble in aqua regia digestion, but had only *ca*. 0.1% of the arsenic and lead content of the other PM. Further, in comparison to the other geodusts, the PM_ES_ were depleted in arsenic and lead with As : Fe and Pb : Fe values of 0.0002 and 0.0026. However, PM_ES_ had almost 10*x* elevated Zn ([Zn]_ES_ = 496 μmol g^−1^) compared to other PM (*e.g.* [Zn]_150_ = 59, [Zn]_SC_ = 46.5 μmol g^−1^) (Fig. S2 and S3†). Despite the greater solubility of efflorescent salts, the arsenic and lead inhalation exposures from PM_ES_ were expected to be lower than other the particles as exposure is a function of total concentration and solubility (Table 1).

**Table 1:** Bioaccessible iron, arsenic, and lead in Iron King Mine Tailings

Sample	[Fe]			[As]		[Pb]	
**Total concentration** a **(mmol·kg** ^**−1**^ **)**							
PM_10_	3330			58.7		34.0	
PM_150_	2220			34.3		11.4	
PM_SC_	1890			38.3		19.0	
PM_ES_	93.1			0.0220		0.0222	
SRM 2782	4820			2.22		2.77	
Background^b^	na			0.297		0.101	

**Molecular speciation** c							
	fh	jar	pyt^■^/cop*	As^V^-fh	As^V^-jar	Pb-jar	ang
PM_10_	55	41	3^■^	24	76	86	14
PM_150_	30	61	8^■^	38	62	71	29
PM_SC_	39	52	10^■^	46	54	na	
PM_ES_	26	39	35*	<DL		<DL	

**Maximum percent BAc lung** d							
PM_10_	1.55^1^			3.64^2^		0.016^1^	
PM_150_	0.15^3^			1.31^2^		0.022^2^	
PM_SC_	0.12^4^			0.37^5^		0.02^5^	
PM_ES_	28.3^4^			2.3^4^		67.5^6^	
SRM 2782	na			1.1^2^		<0.01	

**Percent BAc lung 24 h**							
PM_10_	0.54^a^			1.16^d^		0.003^g^	
PM_150_	0.07^b^			0.74^e^		0.003^g^	
PM_SC_	0.07^b^			0.22^f^		0.006^g^	
PM_ES_	2.3^c^			0.74^e^		7.8^h^	

**Maximum percent BAc gastric** d							
PM_10_	19.1^7^			11.7^7^		3.00^7^	
PM_150_	32.9^7^			25.3^7^		18.2^7^	
PM_SC_	5.8^5^			13.3^5^		0.87^8^	
PM_ES_	93.3^5^			87.9^5^		86.3^5^	
SRM 2782	na			25.4^7^		92.5^7^	

**Percent BAc gastric 1 h**							
PM_10_	2.9^i^			7.8^l^		0.77^o^	
PM_150_	3.6^i^			9.9^l^		0.66^o^	
PM_SC_	1.1^j^			4.3^m^		0.11^p^	
PM_ES_	76.7^k^			44.9^n^		74.5^q^	

a Total concentration in PM_10_, PM_150_, and PM_SC_ determined by Li_2_B_4_O_7_/LiBO_2_ fusion with ICP-MS detection, PM_ES_ total concentrations determined by aqua regia digest with ICP-MS detection, SRM 2782 is the assigned NIST value.

b Background is the site-specific level.^73^

c Molecular speciation of each element as determined by XAS, ^a–q^ BAc significantly different at *p* < 0.05 for 24 h SLF and 1 h SGF.

d Maximum BAc at specified time points: ^1^30 s, ^2^168 h (7d), ^3^5 min, ^4^0.5 h, ^5^48 h, ^6^1 h, ^7^100 h, ^8^24 h; fh = ferrihydrite, jar = jarosite, pyt^■^ = pyrite (from Hayes *et al.*, 2015), ang = anglesite, cop* = copiapite, <DL = less than detection limit, na = not applicable, IVBA results reported are average of triplicates.

Major crystalline phases in PM_10_, PM_150,_ and PM_SC_ determined by synchrotron XRD were quartz [SiO_2_], albite [NaAlSi_3_O_8_], gypsum [CaSO_4_·2H_2_O], pyrite [FeS_2_], jarosite [KFe_3_(SO_4_)_2_(OH)_6_], and chlorite [(Mg,Fe)_5_Al_2_Si_3_O_10_(OH)_8_] (Table 1). Crystalline arsenic or lead phases were not detected by XRD (detection limit ∼2 wt%). Efflorescent salts (PM_ES_) comprised gypsum, magnesiocopiapite [MgFe^(III)^_4_(SO_4_)_6_(OH)_2_·20H_2_O], goslarite [ZnSO_4_·7H_2_O], copiapite [Fe^(II)^Fe^(III)^_4_(SO_4_)_6_(OH)_2_…20H_2_O], hydrated ferric sulfate salts [Fe_2_(SO_4_)_3_·xH_2_O], ferrihydrite [Fe_2_O_3_·5H_2_O], and jarosite. The effective enrichment of metal(loid)s in the finest particulates was due to depletion of coarser primary silicate minerals in the fine size fractions.

### 3.2 Lung fluid IVBA is

#### 3.2.1 Kinetics

**Fig. 1:**
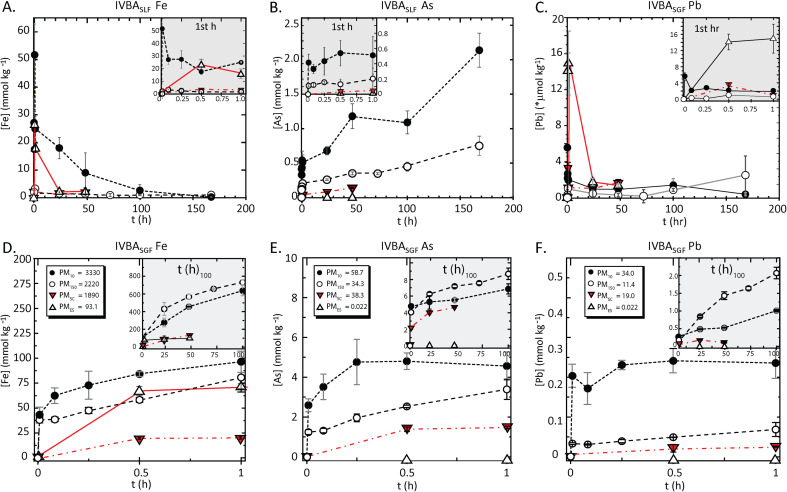
Kinetic bioaccessibility of iron, arsenic, and lead from particulate matter (PM) in synthetic lung and gastric fluids (SLF, SGF). Data shows moles released to solution per unit mass of solid PM (mmol kg^−1^, *Pb SLF is μmol kg^−1^) for iron (left), arsenic (center) and lead (right) from PM_10_ (solid circle), PM_150_ (open circle), PM_SC_ (downward triangle), PM_ES_ (open triangle), in synthetic lung (A–C) fluid to 168 h and gastric (D–F) fluid to 1 h. Inset box details the <1 h interval for SLF and >1 h for SGF. Initial PM concentrations (mmol kg^−1^) are shown above and given in Table 1.

The fractional releases of arsenic and lead, and the dissolution of host ferric minerals were interrogated with a circumneutral SLF to assess *in vitro* kinetic exposure representing inhalation of PM, where elemental release to the biofluid simulant represented the BAc fraction relative to the total PM concentration. Synthetic lung fluid IVBA showed generally low BAc for metal(loid)s in each PM compared to gastric exposures (about 1–10%) (Table 1), with the greatest relative release occurring at the shortest time-points (*e.g.*, minutes) (Fig. 1). Following this rapid pharmacokinetic release, in the closed IVBA batch reaction, iron and lead were observed to repartition back to the solid phase at longer exposures (>24 h). Arsenic showed release to SLF from PM_10_, PM_150_ and PM_SC_, increasing at extended times, whereas PM_ES_ at 48 h showed no significant difference from the maximum release at 5 min. The BET SSA for PM_150_ increased from 2.3 m^2^ g^−1^ (unreacted) to 9.6 m^2^ g^−1^ after 48 h SLF as higher specific surface area particles or mineral coatings precipitated in the lung fluid.

The PM_10_, which showed approximately 2*x* enrichment of arsenic and lead relative to the PM_150_ and PM_SC_ and over 2000*x* enrichment relative to PM_ES_, had a maximum arsenic BAc of 3.6% compared to 2.2% for PM_150_, 0.37% in PM_SC_, and 2.3% in PM_ES_ (Table 1). Lead BAc was not significantly different (*p* > 0.05) for PM_10_, PM_150,_ and PM_SC_ at 0.02%, but 67.5% in PM_ES_.

At 24 h in SLF, the generally used IVBA time point for lung exposure, the arsenic released from the PM_10_ was more than 2*x* PM_150_, and the BAc was significantly larger (*p* < 0.05) than each of the other PM. The lead BAc was low and not significantly different among PM_10_, PM_150_ and PM_SC_; the release at 24 h from PM_ES_ was the highest BAc (7.8%), and was three orders-of-magnitude greater than the other PM. For lead and iron, the maximum release was at the shortest time points (<1 h). Whereas the arsenic and lead mass concentrations (mg kg^−1^, ppm) in individual PM particles were nearly equal, the mole fraction release of arsenic to SLF was about 150*x* higher than that of lead in PM_10_ and PM_150_ and 20*x* PM_SC_; while PM_ES_ showed lead BAc almost 30x greater than arsenic.

#### 3.2.2 Speciation

Micrographs of unreacted PM_ES_ by SEM showed particles with an effective spherical diameter of less than 50 μm and abundant large (25–50 μm) elongated prismatic columns of gypsum; post bioassay imaging was not possible as soluble salts were mostly dissolved *in vitro* (Fig. S4†). Morphological changes were observed by SEM-EDS in the unreacted compared to SLF-reacted PM_10_ and PM_150_ (Fig. 3, S4 and S5†), which showed electron-transparent (*i.e.*, relatively low density) 4–5 μm long acicular neo-precipitates (Fig. S6†). Comparing normalized (to quartz *hkl*_112_) X-ray diffractograms (Cu Kα) of 48 h SLF-reacted PM_150_ to the unreacted PM_150_ showed a new broad feature centered at 20° 2*θ* (4.13 Å) and a new sharp reflection at 13.5° 2*θ* (6.4 Å)(Fig. S7†). The broad feature was attributed to newly formed hydrous ferric oxide (HFO) or ferrihydrite (PM_150_logΩ_24h_ = 4.4; calculated from the aqueous chemistry of PM_150_ at 24 h in SLF), while the sharp reflection at ∼6.4 Å was a fair fit (with multiple overlapping peaks) to the calcium sulfate hemihydrate mineral bassanite (CaSO_4_·½H_2_O), consistent with the micrograph images of acicular or columnar crystals (Fig. S6†). Pyromorphite, an expected sink for lead in high phosphate environments, with a maximum reflection at 30.5° 2*θ* (100% *hkl*_112_ = 2.92 Å), was obscured by an overlapped jarosite peak at 30.14^o^ 2θ (10% *hkl*_202_ = 2.97 Å) in PM_10_ and PM_150_ (Fig. S7 and S8†). Mineralogical changes in PM_10_ were less apparent in XRD diffractograms, except increased relative intensity of peaks from gypsum post SLF IVBA.

##### 3.2.2.1 Iron species

**Fig. 2:**
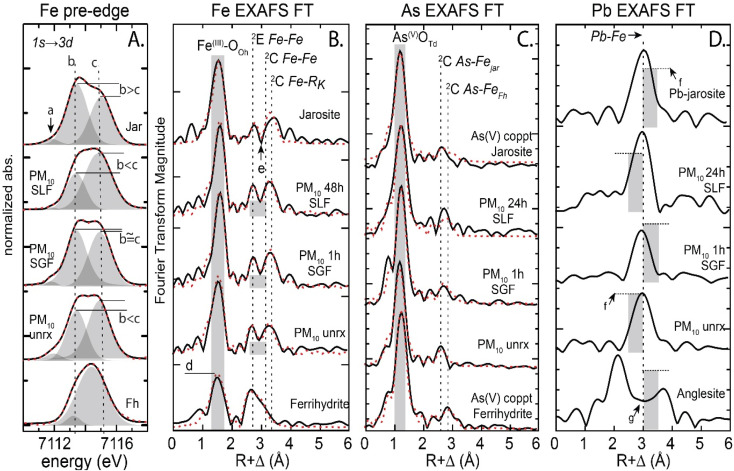
Iron XANES pre-edge and iron, arsenic, and lead EXAFS Fourier transform of unreacted and IVBA reacted PM_10_ from Iron King mine tailings. Panel A shows Fe XANES pre-edge, with peaks ^*a*–*c*^ characteristic of ferrihydrite v jarosite in oxidized sulfidic tailings. Panel B shows the EXAFS FT for PM_10_ reacted in lung and gastric fluid *in vitro*, ^*d*^indicates Fe–O backscattering and highlights FWHM peak change for ferrihydrite v jarosite and ^*e*^indicates separation of edge sharing Fe–S/Fe and corner sharing Fe–Fe in ferrihydrite and jarosite, fits shown in Table S2.† Panel C shows As EXFAS FT with first shell As–O at 1.68 ± 0.02 Å and second shell As–Fe Δ0.02 Å. Panel D shows Pb EXFAS FT, ^*f*^Pb–Fe FT peak amplitude is greater for PM_10_ unrx. ^*g*^indicates region of FT where Pb–O features would be observed for Pb-sorbed ferrihydrite.

Evaluation of the PM_10_ Fe Kα XANES pre-edge (7110–7118 eV), a feature resulting from an allowed transition of the 1s electron to a 3d orbital, showed slight changes in the three assigned peaks (*a-c*) that make up the pre-edge (Fig. 2A). The lowest energy peak (*a*) at 7112 eV was present in jarosite and absent in ferrihydrite. The *a* peak decreased in the 48 h SLF reacted sample compared to the unreacted PM_10_ particles. Additionally, the subsequent pre-edge peaks (*b*) at 7113 eV and (*c*) at 7115 eV show a relative increase in *c*, over *b*, post IVBA. The pre-edge in the PM_150_ showed slight changes in the three assigned peaks (*a*–*c*), similar to PM_10_ with the (*a*) peak diminished in SLF at 48 h (Fig. S9†). Linear combination fits (LCF) to the PM_10_ Fe EXAFS showed that the iron in the unreacted tailings was comprised of jarosite (41%), ferrihydrite (55%) and pyrite (3%), PM_150_ was jarosite (61%), ferrihydrite (30%) and pyrite (8%), PM_SC_ had, jarosite (52%), ferrihydrite (39%), and pyrite (10%), and unreacted PM_ES_ was jarosite (39%), ferrihydrite (26%), and copiapite (35%). Iron EXAFS of unreacted and 48 h post IVBA PM tailings were compared. The 1^st^ shell feature in the Fourier transformed Fe Kα EXAFS, attributed to the Fe^III^–O_6_ backscattering, increased in amplitude for the 48 h SLF-reacted PM_10_ relative to the unreacted PM_10_ and the Fe–O radial distance increased from 1.94 Å to 1.97 Å (Fig. 2B and S10,Table S2†). Second shell contributions for near neighbor iron backscattering atoms varied with the contribution of jarosite and ferrihydrite, and fits were constrained with a binary structural model for fitting. Backscatterer Fe–Fe distances varied by the major contribution from the identified species, where longer corner sharing Fe–Fe distances at 3.64 ± 0.01 Å and Fe–S at 3.24 ± 0.01 Å were assigned to jarosite and shorter Fe–Fe distances of 3.07 ± 0.02 Å and 3.36 ± 0.01 Å were assigned to ferrihydrite. At 1 h, PM_10_ showed a decrease by about 50% in coordination of the Fe–Fe at 3.08 ± 0.1 Å and 3.35 ± 0.1 Å from 1.5 and 3.1 to 0.7 and 1.4, respectively (Table S2†). These distances were attributed to the contribution from Fe–Fe backscattering from edge-sharing (^2^E) and corner-sharing (^2^C) Fe^III^ octahedra in ferrihydrite, respectively.^74^ The Fe second shell contribution showed a decrease in the contribution from ferrihydrite at 1 h and longer, *e.g.*,48 h (Table S2†). No significant changes were observed in the jarosite associated backscattering near neighbors. The post SLF IVBA PM_150_ Fe EXAFS showed small decreases in coordination of the Fe–S and Fe–Fe to 1.4 and 1.2, respectively (Fig. S11 and Table S3†). While the minor change noted in the jarosite associated Fe–Fe backscattering near neighbors at 3.63 Å was within the error of fitting coordination numbers in complex matrices, it is nonetheless consistent with a reduction in the jarosite contribution to the EXAFS signal. Fitting the post IVBA PM_ES_ showed significant alteration of the Fe second shell neighbors with a depletion in Fe–S at 3.27 Å and alteration of the Fe–Fe 3.26 Å and 3.40 Å distances associated with ferrihydrite (Table S4†). The complex dissolution and reprecipitation in the PM_ES_ is not resolvable by Fe EXAFS but does indicate a loss of Fe–S second shell ligand, likely attributed to dissolution and loss of soluble ferric sulfate salts and jarosite.

##### 3.2.2.2 Sulfur species

The unreacted PM_10_ sulfur speciation was dominated by gypsum (49.1%), which was depleted by extended incubation *in vitro* (Table S5, Fig S12 and S13†). The relative contribution of pyrite to the sulfur XANES increased with each time step, as sulfate species were solubilized. Other components of the fits increased concomitantly, with the relative contribution of pyrite doubling, and the jarosite signal increasing slightly less than the ferrihydrite-adsorbed sulfate contribution. Sulfur speciation in unreacted PM_150_ was mostly jarosite (60%), and at long reaction times 7 d in the SLF the contribution from pyrite was unchanged, jarosite increased to over 80% of the S signal while gypsum and adsorbed sulfate were depleted relative to other species.

##### 3.2.2.3 Arsenic species

The solid phase arsenic speciation at each kinetic step probed by EXAFS showed As^(V)^–O_4_ in tetrahedral coordination at 1.68 ± 0.02 Å (Table S6 and S7†). The As EXAFS showed changes in second shell ligand distances as a function of the mixing ratio of ferrihydrite and jarosite associated arsenate (Fig. S14†). The spectral contribution from jarosite associated arsenate was greater than arsenic sorbed ferrihydrite in PM_10_, PM_150_, and PM_SC_ and was not detectable in PM_ES_ (Table 1). In the unreacted PM_10_, 76% of the arsenic was associated with jarosite and 24% with ferrihydrite, even though PM_10_ was enriched (with respect to iron) in non-arsenic associated ferrihydrite relative to PM_150_ and PM_SC_ (Table 1). Unreacted PM_10_ had a longer As–Fe distance of 3.32 Å compared to the unreacted PM_150_ and PM_SC_, which was 3.28 Å (Table S6–S8†). Post SLF IVBA, PM_10_ had a slight lengthening of the As–Fe distance from 3.32 Å to 3.34 Å at 48 h. The small 0.02 Å change (>estimated fitting error ±0.015 Å) supports, but is not compelling evidence for, a greater contribution to the EXAFS signal from arsenate complexes with slightly longer As–Fe distances at 48 h and an attendant reduction in the signal from shorter As–Fe bonds (<3.30 Å). The PM_150_ As EXAFS showed a lengthening of the As–Fe second shell ligand at 48 h to 3.32 Å, compared to the unreacted PM_150_ at 3.28 Å.

##### 3.2.2.4 Lead species

The unreacted PM_10_ and PM_150_ lead speciation was best described by two species, plumbojarosite and anglesite. The PM_10_ particles were enriched (86%) in plumbojarosite relative to PM_150_ (71%) (Table 1). Reaction in SLF showed no significant change in lead speciation for PM_10_, while PM_150_ showed a decrease in plumbojarosite and consequent increase in anglesite over 7d *in vitro* (Table S9 and Fig S15†).

### 3.3 Gastric fluid IVBA

Exposure risk from ingestion of metal(loid)s from IK tailings was interrogated with acidified 0.4 M glycine at pH 1.5 as a gastric fluid surrogate. The ≤1 h exposures of PM to SGF represents biorelevant durations of residence times expected in the GI track and may better represent *in vivo* exposures compared to long incubations^46^ (Fig. 1 and Table 1). However, while the long duration SGF assays (>1 h) may not represent realistic *in vivo* residence times, these nonetheless indicated mechanisms of dissolution and reprecipitation that could occur with extended bioassay or physiological mechanisms not mimicked *in vitro*. Synthetic gastric IVBA showed generally higher BAc for each element in each PM relative to synthetic lung fluid IVBA.

#### 3.3.1 Kinetics

The metal(loid) release rate to synthetic gastric fluid from each PM was greatest at the early intervals. For example, a parabolic release curve was observed with arsenic reaching 8.2% and 9.9% after just 0.25 h for PM_10_ and PM_150_, respectively (Fig. 1). The release of total metal(loid)s from PM_10_ was greater than other PM for time steps ≤ 1 h, but at 100 h PM_150_ had the greatest release. After the first hour, the asymptotic release of arsenic approached a BAc of 10% PM_10_, 25% for PM_150_, 13% for PM_SC_, and nearly 90% for PM_ES_ (Fig. 1 and Table 1). The iron and arsenic released from PM_SC_ showed an increase at each successive step to 48 h where 5.8% and 13.3% of the total iron and arsenic, respectively was dissolved, a trend similar to the PM_10_ and PM_150_. Lead released from PM_SC_ had a maximum at 24 h and a return to the solid phase at 48 h. Release kinetics from PM_ES_ showed a relative increase at each successive interval to termination at 48 h, where 93.2%, 87.9%, and 86.3% of the iron, arsenic and lead were released, respectively. The BET SSA for PM_150_ decreased from 2.3 m^2^ g^−1^ (unreacted) to 1.6 m^2^ g^−1^ after 1 h in SGF (and 1.1 m^2^ g^−1^ after 48 h), indicating dissolution of higher surface area particles in the acidic fluid.

#### 3.3.2 Speciation

**Fig. 3:**
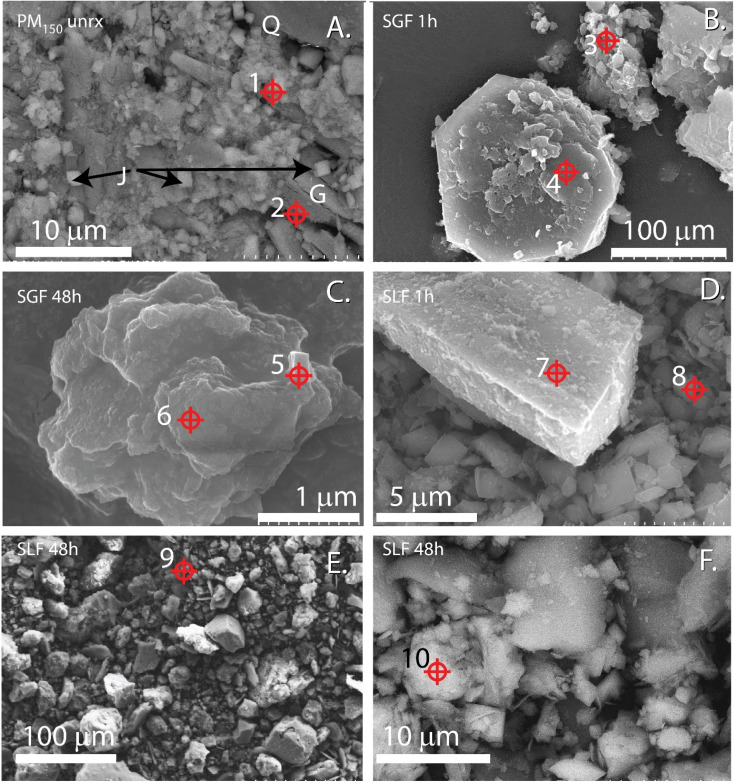
Electron micrographs of <150 μm effective spherical diameter particulate matter (PM_150_) isolated from IKMHSS tailings unreacted (A) and after *in vitro* gastric simulant bioassay for 1 hour (B), and 48 hours (C), and lung fluid for 1 h (D) and 48 h (E and F). Crosshairs indicate EDS spots,^1–10^ spectra given in Fig. S5.† Panel (A) J = jarosite, Q = quartz, G = gypsum. Scale bar indicates magnification.

Relative to the unreacted PM, the post SGF IVBA XRD diffractograms showed a relative decrease in the jarosite signal and nearly unchanged pyrite peaks, and a flattening of the lower 2*θ* baseline attributed to loss of amorphous and short-range order ferric hydroxides or ferrihydrite (Fig. S7 and S8†). Morphological changes were observed in SEM micrographs of the unreacted PM_150_ tailings compared to 1 h in SGF (Fig. 3). At 1 h in SGF, rough surface texture is reduced, and at extended times (48 h) grains have lost the fine texture (Fig. 3 A–C).

##### 3.3.2.1 Iron species

Linear combination fits (LCF) to the Fe EXAFS for unreacted tailings are given (Table 1). Shell by shell fitting showed PM_10_ reaction in SGF for 1 h resulted in about 50% diminished contribution of ferrihydrite as shown in the Fe–Fe distances at 3.08 Å and 3.35 Å, and this effect increased at extended reaction times (Table S2†). Additionally, the Fe EXAFS FT showed an amplitude increase and narrowing of the Fe–O in the first shell peak after 1 h reaction in SGF (Fig. 2). There was no significant change in PM_150_ at 3.08 Å but a 25% diminished contribution and 0.04 Å reduction in distance from 3.38 Å to 3.34 Å in the longer Fe–Fe after 1 h reaction in SGF (Table S3†). The changes noted in the ferrihydrite associated Fe–Fe backscattering near neighbors at approximately 3.04 Å and 3.34 Å were consistent with a reduction in the ferrihydrite to the EXAFS signal for PM_10_ and PM_150_. The jarosite contribution to the EXAFS spectra from PM_10_ showed no significant change in the coordination number or radial distance of the Fe backscatters after 1 h in SGF (Table S2†). At 48 h, the PM_10_ jarosite contribution showed a reduction in coordination of the Fe–Fe corner sharing octahedra at 3.63 Å of about 3.2 to 2.0 and 1.6 to 1.0 for Fe–S at 3.27 Å. Insufficient PM_ES_ remained for XAS after IVBA in SGF.

##### 3.3.2.2 Sulfur species

Similar to SLF, reaction in the acidic SGF showed the PM_10_ and PM_150_ contribution from gypsum to the sulfur XANES spectra decreased at each interval *in vitro* (Table S5 and Fig S12†), and the contribution of pyrite to the sulfur XANES increased relative to the unreacted PM as the sulfate species were solubilized (Fig. S13†). At the longest incubation *in vitro*, the sulfur XANES signal was dominated by jarosite in PM_10_ and PM_150_, as other species dissolved.

##### 3.3.2.3 Arsenic species

The arsenic speciation, determined to be arsenate, did not change during SGF (or SLF) IVBA (Table S6 and S7†). Second shell As–Fe backscattering at 3.28 Å in PM_150_ increased slightly in coordination number from 1.5 to 2.2 at 1 h. The second shell As–Fe distance in PM_10_ shortened post 1 h SGF, from 3.32 Å to 3.30 Å at 1 h and 3.29 Å at 24 h, indicating preferential removal of longer range As–Fe. The As EXAFS also showed changes in second shell ligand distances of As–Fe as a function of the mixing ratio of ferrihydrite and jarosite associated arsenate.

##### 3.3.2.4 Lead species

Analysis of Pb-L_III_ EXAFS indicated that PM_10_ contained lead mostly in plumbojarosite, with good alignment of backscattering oscillations in *k* space (Å^−1^) but a reduced amplitude in the χ(k)·k^3^ EXAFS compared to reference plumbojarosite (Fig. S15†). The amplitude reduction was attributed to out of phase lead -bearing solids *e.g.* anglesite, consistent with previously reported tailings materials.^20^ Reaction in SGF showed a decrease in the contribution of plumbojarosite for PM_10_ and PM_150_ after 1 h. (Table S9†).

## 4 Discussion

Fugitive dusts in arid and semi-arid environments exhibit hygroscopic growth *in vivo* under conditions of high humidity that enhance alveolar deposition and inhibit lung clearance.^76^ When particles finer than 10 μm effective spherical diameter (PM_10_) pass the tracheobronchial system into the lungs, the relative solubilities of the remaining compounds will dictate what dissolves into extracellular fluids, while insoluble compounds can accumulate as particles in the lungs.^77^ Larger particles (>PM_10_) that do not pass into the respiratory regions of the lung are trapped in mucus by cilia as part of the body's filtration defense. The muco-ciliary escalator mechanism clears larger particles from the airway toward the pharynx, where they are swallowed and become susceptible to gastric fluid dissolution.^78^

The maximum mass release of toxins in synthetic lung fluid at ≤24 h (Table 1), and the extrapolated risk to human health from inhalation exposures, from Iron King mine tailings particles for arsenic was PM_10_ > PM_150_ > PM_SC_ > PM_ES_ and for lead was PM_ES_ > PM_10_ > PM_SC_ > PM_150_. The pharmacokinetic release to SLF from PM_10_ shows greatest risk at short duration exposures (<0.5 h) for lead and a delayed exposure risk for arsenic, with the greatest arsenic release measured in this study at the longest time step (Fig. 1). Combining sorbate elemental analysis with sorbent multi-element XAS, XRD, SEM and BET at several bio-relevant kinetic steps indicated arsenic-substituted jarosite was the most bioaccessible species with respect to arsenic, and the toxic metalloid host phase posing the most risk in lung fluid. Precipitation of arsenic or lead bearing minerals in lung fluid can remove a contaminant from the dissolved phase, but it is unclear the extent to which such *in vivo* lung fluids achieve supersaturation and, if so, neoformed particles may also act as irritants and long-term point-sources of chronic exposure at extended residence times (*e.g.*, years). However, in lung tissue, the larger volume of fluid and continuous throughflux of new fluid interacting per unit mass of particle is likely to preclude, to some degree, the back reaction (*i.e.*, secondary precipitation formation).

### 4.1 Arsenic speciation

In unreacted PM, arsenic was distributed between ferrihydrite and jarosite (Table 1 and Fig. S14†). The surface crust had the highest ferrihydrite associated arsenic, lowest jarosite associated arsenic, and the lowest 24 h BAc in lung fluid, 0.22% (and lowest maximum 0.37% at 48 h) and lowest in gastric fluid, 4.3% at <1 h. With increased content of short-range ordered minerals like ferrihydrite in metalliferous-mine tailings, arsenic shows a corresponding decrease in bioaccessibility for SGF and SLF, attributable to the high activity of high-affinity sites for surface complexation of arsenate.^45^

The reported EXAFS determined As–Fe interatomic distances in jarosites are: 3.25–3.26 Å for arsenic co-precipitated jarosite;^79–81^ 3.35 Å for high arsenic jarosite loadings (17 mole%, possible contribution from scorodite);^75,80,82^ and 3.22 Å for ^2^C coordination of As-adsorbed jarosite.^79^ Ferrihydrite with low to moderate arsenic loading (Fe : As > 50) displays As–Fe ^2^C coordination at 3.28 Å for bidentate arsenate tetrahedra corner-sharing oxygens with two edge linked iron octahedra, as determined by EXAFS and DFT calculations.^74^ These distances are shorter than the longer distances of 3.32 Å (^2^C) reported for colloidal amorphous ferric arsenate.^74,83^ Arsenic-rich amorphous particles have been reported to occur as highly-aggregated clusters, 10 to 100 nm in size, composed of corner-linked iron octahedra in bidentate-binuclear complexation with arsenate.^84^ While the arsenic in the PM was distributed between surface complexed arsenate on ferrihydrite and substituted for sulfate in jarosite, the long As–Fe distance of 3.30–3.34 Å observed in the unreacted PM_10_ indicated arsenic was also associated with colloidal amorphous ferric arsenate in ^2^C coordination,^84^ whereas the relatively shorter As–Fe distances in PM_150_ of 3.27–3.28 Å are in agreement with bidentate binuclear arsenic adsorbed on ferrihydrite reactive surface sites.^74^ The PM_10_ displayed relatively longer As–Fe bonds (>3.30 Å), consistent with high arsenic loading of ferric hydroxide octahedra. Whether high arsenic ferric solids were linked chains^80^ or ferric arsenate clusters,^83^ is beyond the scope of this study.^74^

#### 4.1.1 SGF

Gastric IVBA carried out to 100 hours approaches an extreme exposure duration. Here, the 1 h and less duration exposures are emphasized as those that are physiologically relevant (Fig. 1). However, long kinetic steps were applied to explore positive and negative interferences in toxin release from competing reactions and dissolution mechanisms *in vitro* that may not mimic *in vivo* physiology. In contrast to SLF, changes in the post SGF atomic coordination of arsenic showed slight shortening of the As–Fe distance from 3.32 Å (PM_10_) and 3.28 Å (PM_150_) to 3.28 Å and 3.27 Å, respectively, consistent with a reduction in the contribution of arsenate coprecipitated with ferrihydrite 3.28 Å^74^ and an increase in the contribution of the shorter distance As–Fe jarosite component of 3.27 Å.^75,80^ Arsenic liberated from surface complexation sites on ferrihydrite was the major contributor to the SGF BAc at initial time points, and at extended incubations *in vitro* arsenic may partition back to the solid phase in As-jarosite or Pb–As-jarosite, or as a surface complex on freshly precipitated β-FeOOH (PM_150_ log *Ω*_100h_ = 2.43). Although neo-precipitate ferric hydroxides were observed to be supersaturated in the closed and extended SGF IVBA, they would not be expected to form in the gastrointestinal tract *in vivo* and would therefore not likely serve as a sink for dissolved arsenic in a physiological system. However, under the acid conditions in gastric fluid, arsenic released from soluble phases like calcium arsenate or arseniosiderite could adsorb to co-occurring, sparingly-soluble ferric hydroxides thus attenuating the measured bioaccessible fraction.^85,86^ At the acid pH of SGF (1.5), precipitation of ferrihydrite was not thermodynamically favorable (PM_150_ log *Ω*_100 h-SGF_ = −2.2), although there was a thermodynamic drive for akaganeite and plumbojarosite (PM_150_ log *Ω*_100 h_ = 11.3) precipitation. The pre-existence of jarosite surfaces could serve as a template to decrease the activation energy (degree of supersaturation) required for nucleation and crystal growth,^87,88^ which could sequester both arsenate and lead during long term low pH SGF IVBA exposure. Because ingestion exposure to PM_ES_ exhibited rapid solubilization on the time scale of minutes to hours, this suggests that total concentration was a reasonable indicator of BAc of arsenic and lead in efflorescent salts. However, the total arsenic concentration in the PM_ES_ was 3 orders of magnitude lower than the other PM (Table 1). Whereas the 1 h SGF bioaccessible fraction of PM_10_, PM_150_ and PM_SC_ were all much lower (4.3–9.9%) compared to PM_ES_ (44.9%), the total mass of arsenic released per gram of geodust was much lower in the PM_ES_ ([As_ES_]_T_ = 0.022 μmol g^−1^), and therefore PM_ES_ poses a lower IVBA determined risk of arsenic exposure.

#### 4.1.2 SLF

About 20% of the total SLF released arsenic occurred at 30 s and aqueous arsenic concentrations increased in all PM at each kinetic measurement step, releasing about 80% of the BAc arsenic after the initial pulse (Fig. 1). Investigation of SLF arsenic bioaccessibility of PM_20_ from Au mining wastes from southern Australia, where the principal arsenic bearing phase was scorodite (FeAsO_4_·2H_2_O), showed a rapid release followed by a modest increase in arsenic dissolution to 24 h, but in that study total BAc arsenic was <0.1%.^89^ Here, BAc values were more than an order of magnitude higher, indicating that the mixture of arsenate-rich jarosite and ferrihydrite pose a much greater inhalation risk than scorodite dominated tailings. Additionally, studies have shown soil arsenic BAc varies with speciation; BAc showed jarosite > ferrihydrite > scorodite > arsenopyrite.^90^

At extended exposures (>48 h), PM_150_ in SLF resulted in a lengthening of the As–Fe distance consistent with ^2^C coordination of amorphous ferric arsenate. *In silico* calculations indicate atomic coordination similar to the substitution of tetrahedral sulfate by arsenate in the T-Oh_3_ jarosite structure in tridentate penta- and hexa-nuclear complexes.^91^ The substitution of an arsenate tetrahedron (As–O_4_ 1.69 Å) into a sulfate tetrahedron site (S–O_4_ 1.54 Å) in the jarosite crystal system creates torsional and steric strain in the iron octahedral kagome lattice, decreasing the activation energy for breaking the As–O–Fe bonds, which may make arsenic from highly substituted jarosite more susceptible to release *in vivo* than from bidentate binuclear surface complexes at ferric (hydr)oxide surface sites.

**Fig. 4:**
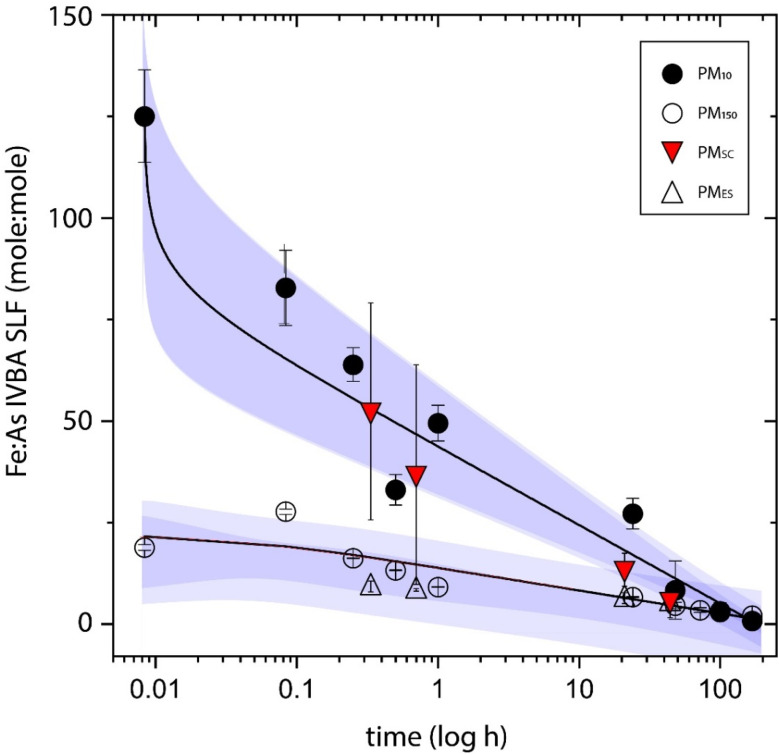
Mole ratio of released iron and arsenic in SLF. Solid circles are PM_10,_ open circles are PM_150_, closed downward red triangles are PM_SC_, open triangles PM_ES_, lines represent the best fit (*y* = *a* − b*ln(*x* + *c*)) to the data, PM_10_*R*^2^ = 0.946, PM_150_, *R*^2^ = 0.829, fits to SC and ES not shown. Dark shaded bands represent the 95% confidence interval, light shading represents the 95% prediction band.

Here, Fe XANES pre-edge combined with iron and arsenic EXAFS indicated that jarosite was the species most susceptible to SLF extraction. The lengthening of the 3.32 Å As–Fe bond in post SLF solids at 48 h further indicates a relative loss of the shorter As–Fe distance from As-loaded jarosite and/or accretion of arsenic complexed with ferric hydroxides. The affinity of arsenate for adsorption to ferric (hydr)oxide surfaces generates a surface complex that is relatively resistant to SLF IVBA. The high activity of phosphate in the SLF and sulfate from the dissolution of soluble salts (*e.g.*, gypsum) contributed competing ions that inhibited arsenic re-sorption, evidenced by S XANES showing increased loading of sulfate on ferrihydtite as the SLF reaction progressed, from 13% to 22% (Table S5†). The release of arsenic by SLF IVBA was parabolic, but not asymptotic, over the duration examined for lung fluid extractions in the PM_10_ and PM_150_ size range (Fig. 1), indicating more than one kinetic reaction rate, with an initial release dominated by dissolution of strained arsenic-substituted jarosite and secondary competing processes of solid phase repartitioning or precipitation under saturated conditions. It should be considered that the observed parabolic release may be due to accumulation of solutes in the closed IVBA batch system that would not develop in an open biosystem with continuously exchanging fresh biofluid. The release of arsenic from PM in SLF was not stoichiometric with iron. The ratio of Fe : As in unreacted PM_10_ and PM_150_ solids was 56.7 and 64.7, respectively. At the first SLF extraction step, the PM_10_ release was dominated by iron with respect to arsenic, 30 s Fe : As = 124, while PM_150_ showed diminished iron release relative to arsenic, Fe : As = 18.2 (Fig. 4). Both particle types showed an enrichment of arsenic release relative to iron release with increased reaction times, and the release of arsenic in both PM_10_ and PM_150_ to SLF approached unity (PM_10_ Fe : As_100h_ = 2.13, PM_150_ Fe : As_100h_ = 2.27) with extended incubations (Fig. 4). The Fe : As ratio in the solubilized (bioaccessible) pool decreased at each interval in PM_10_ and PM_150_ and fit to non-linear natural log of time curve (PM_10_*R*^2^ = 0.946, PM_150_*R*^2^ = 0.829) (Fig. 4). Where the molar ratio of Fe : As released to SLF was lower than the bulk ratio, *i.e.* after 15 minutes, for PM_10_ and over the duration of the IVBA experiment for PM_150,_ indicated that (i) dissolution of an arsenic enriched species, (ii) preferential arsenic release, or (iii) reprecipitation of iron were responsible for the solubilized species; and where the arsenic release exceeded iron it was seen as evidence that iron was precipitating as ferric (hydr)oxide without concurrent arsenic sorption.

### 4.2 Lead speciation

#### 4.2.1 SGF

The release of lead from PM_10_ to SGF increased with time to 100 h, and BAc reached a maximum of 18.2% (Fig. 1). The iron gastric IVBA followed a release curve very similar to lead (and arsenic), whereby it increased at each step to 100 h. The initial, a rapid, release of lead from the tailings was attributed to lead sequestered in acid soluble anglesite, which was released early in the reaction. Equilibrium modeling of anglesite solubility at the low pH and geochemical conditions of the SGF show a drive toward instability (log *Ω*_100h-SGF_ = −17.1), which would release lead to gastric fluid.^92^ Whereas equilibrium modeling showed the potential for precipitation of akaganeite, at the low pH of SGF Pb^2+^ sorption would be minimized and would not serve as a sink for lead. Further, because of the low phosphate activity and low pH, precipitation of pyromorphite as a sink for lead was not expected in SGF.^92,93^ Lead released to the aqueous phase at extended reaction times could be limited by the stability of plumbojarosite (PM_10_ log *Ω*_100h-SGF_ = 10.9), for which the 100 h solution was supersaturated and could promote sequestration of lead (and iron) into the solid phase. The low pH SGF solution was not supersaturated with respect to any other lead minerals. Therefore, it is not expected that any neo-phase provided a sink for Pb^2+^ at extended exposures.

#### 4.2.2 SLF

The maximum lead released in PM_10_ SLF occurred very quickly (less than 30 s), and the release at very short intervals suggests dissolution of a sulfate salt like anglesite (PbSO_4_). The removal of lead from the aqueous phase after the initial pulse to SLF was attributed to supersaturation with respect to lead bearing apatite group minerals, *e.g.* pyromorphite ([Pb_5_(PO_4_)_3_(Cl,OH)], PM_10_ log *Ω*_24h-SLF_ = 6.9) and subsequent precipitation. In the SLF, plumbo-jarosite [Pb_0.5_Fe_3_(SO_4_)_2_(OH)_6_] precipitation was also predicted (PM_10_ log *Ω*_24h_ = 17.6). Meanwhile, solutions were near equilibrium with respect to jarosite (PM_10_ log *Ω*_24h_ = 0.55). The Fe : Pb ratio in unreacted PM_10_ solids was 98.0 (Table 1). The solubilized BAc Fe : Pb ratio ranged from 4.4–16 for all times through 100 h, indicating that the drive for dissolution and repartitioning of iron to the solid phase was much greater than for lead under high dissolved solids and circumneutral conditions of SLF. Lead XAS showed very little change in either XANES or EXAFS between unreacted and extended time points of SLF-reacted PM_150_ (Fig. S15 and Table S9†). The bioaccessible fraction of lead was very low, and spectroscopic evidence points to anglesite, a biofluid soluble lead-sulfate salt,^94^ consistent with the observed kinetic lead release at seconds to minutes.^20^ The potential inhalation risk to lead exposure from IK mine particles was greatest for the PM_ES_, which had 100*x* lower total lead concentration compared to PM_10_ but released almost 68% in 48 h to SLF. The rapid-release of lead to SLF was likely due to the dissolution of anglesite, or lead (Pb^2+^) substituted zinc (Zn^2+^) sulfates. It has been shown that the BAc lead in mine tailings increases with increased weathering, where sulfides (galena, PbS) in the absence of excess PO_4_^3−^ transform to plumbojarosite, and where pH conditions are not sufficiently low to promote the precipitation of plumbojarosite, anglesite forms as evaporative efflorescent salts and poses great inhalation hazard.^20,94^ Efflorescent salts in and on mine tailings in dry climates may pose greater human health risk of lead exposure relative to humid environments precisely because of the enhanced formation of efflorescent salts and increased offsite transport of aerosols.^2^ Specifically, the weathering of mine tailings in arid regions induces unique hydrogeochemically driven diagenetic alterations that impact the health risk of particulate exposures, attributed to evaporative deposition of lead-containing PM_ES_ at the surface.^15,20,94^

### 4.3 Iron and sulfur speciation

The risk associated with inhalation of PM, and subsequent exposure to arsenic and lead in the lungs from weathered sulfidic mine tailings, has been shown to be lower than gastric exposure at physiologically relevant time points of 1 h for SGF and 24 h for SLF. Further, health risks associated with arsenic PM exposure were mitigated, it seems, as arsenic BAc decreased with an increase in amorphous iron as ferrihydrite (Table 1). Iron release to the circumneutral pH and oxic conditions of SLF induces solution phase supersaturation with respect to ferrihydrite (PM_150_ log *Ω*_24h_ = 4.4). The precipitation of ferrihydrite would provide fresh protonated surface sites (≡FeOH_2_^+^) for sorption of aqueous ions, *e.g.* oxyanion As^5+^ (H_*x*_AsO_4_^3−*x*^, p*K*_a_^*x*=1^ = 6.96). The strong affinity of arsenate for ferric hydroxide surfaces from neutral to acid pH can partially explain the low net release of arsenic to SLF, where arsenate forms inner-sphere bidentate binuclear complexes at ferric hydroxide surface sites.^95^ Whereas ferrihydrite was not expected to precipitate in the low pH SGF (PM_150_ log *Ω*_100 h-SGF_ = −2.28), the modeled iron speciation of PM_150_ showed ferric hydroxy-chloride, similar to akaganeite (β-FeOOH), was supersaturated at long reaction timesteps in SGF. Precipitation of amorphous ferric hydroxides, inclusive of ferrihydrite and akaganeite, could scavenge oxyanions *in vitro*,^34^ but would not sorb Pb^2+^ below pH 3.^96^ Additionally, Fe XANES and EXAFS showed no evidence of increased ferric hydroxides in SGF at the longer residence time (Fig. 2). Second shell backscattering near neighbors of Fe–Fe in edge sharing coordination assigned to be associated with ferrihydrite showed a shortening of the Fe–Fe distance at 3.04 Å to 3.02 Å and 3.38 Å to 3.34 Å after 1 h in SGF. Longer distance contributions of Fe–Fe from corner sharing octahedra at 3.62 ± 2 Å showed a decrease in coordination from 2.3 to 1.0, and an insignificant increase in radial distance from 3.62 Å to 3.63 Å, and no change was observed from the contribution of Fe–S backscattering, but since no change was noted in the number of backscattering S atoms from 3.24 Å, dissolution of the jarosite component was not supported. An amorphous ferric arsenate structural model (‘chain model’) with Fe–Fe distances of about 3.6 Å has been proposed for sulfide mine waste environments and, if present, would partially explain the reduction in coordination of longer Fe–Fe 3.62 Å without a reduction in the Fe–S from jarosite.^97^

It was determined that the diminished soluble iron at the longest SLF exposures was due to *in vitro* artifact ferric solid precipitation. The PM_10_ was the particle of greatest inhalation risk to arsenic exposure, having both the highest total concentration and the greatest BAc release to SLF. The arsenic released to lung fluid for all particles (in decreasing concentration) was 2.1 μmol g^−1^ for PM_10_, 0.76 μmol g^−1^ for PM_150_, 0.16 μmol g^−1^ for PM_SC_ and 0.5 nmol g^−1^ PM_ES._ This becomes problematic as fine particles lodged in the lung could reside for days to years, and SLF IVBA showed increased risk to arsenic exposure from increased residence time *in vivo*. In addition to toxic response from arsenic and lead, oxidized iron particles can cause cell damage as they can act as electron shuttles (Fenton-like reactions) and produce oxidants and electrophiles that induce inflammation and oxidative stress.^98^

The high dissolved-salt and circumneutral environment of the SLF batch IVBA favored the precipitation of new solid species. Bioaccessibility release kinetics indicate that lead and iron partitioned back to the solid phase after an initial release to solution (Fig. 1), potentially as pyromorphite and hydrous ferric oxide. These precipitates had a lower affinity for arsenic than for lead, as indicated by the continued release of arsenic relative to lead over time. Hence, the low Pb_SLF_ release was attributed to *in vitro* formation of sparingly soluble pyromorphite (PM_150_ log *Ω*_24h_ = 7.7). Sufficient excess PO_4_^3−^ (1 mM) was present, not only to induce pyromorphite precipitation, but also to compete with dissolving AsO4^3−^_(aq)_ for newly generated surface hydroxyl sites of precipitating ferric (hydr)oxide, thereby promoting higher aqueous phase arsenic in SLF. Together with the high phosphate activity, competition from excess sulfate likely also inhibited arsenic re-adsorption.^99^ Although PM_150_ metal(loid) bioaccessibility in SLF was low overall, the nature of PM dissolution incongruency affects As and Pb quite differently, such that these elements exhibit distinct patterns of net release that are strongly dependent on exposure time. While the back reactions promoted by supersaturation of sequestering solids may not be feasible *in vivo*, the mineralogy, speciation and solution chemistry should be evaluated as possible interferences *in vitro*.

In circumneutral lung fluid, at extended contact times (100 h), there was a thermodynamic drive for precipitation of ferrihydrite in SLF. However, as ferrihydrite formation can explain the decrease in aqueous iron activity; high phosphate and sulfate activity promoted ligand exchange and/or inhibited surface complexation of arsenate, resulting in increased arsenic released to lung fluid with each kinetic step from all particles studied. Because the release of arsenic from PM_10_ in lung fluid did not reach steady state during the IVBA, the potential slow release of lodged particles may present a long-term chronic health concern for particles with extended residence time. The risk associated with lead inhalation was greatest from efflorescent salts, while the total concentrations from PM_ES_ used here were relatively lower than other PM, the BAc was much greater. Conversely, the release of lead in SLF was attenuated with longer residence times, attributed to the precipitation of insoluble pyromorphite species.

At the Iron King site in AZ, the tailings have >0.2 wt% arsenic and lead, however analysis indicated that sequestration in jarosite reduced exposure risk of arsenic and lead from fine particles in low pH gastric fluid, consistent with recent findings.^39^ The greatest deleterious health risks from ingestion of fugitive dusts were expected for the PM_10_, which showed multi-metal(loid) enrichment, with arsenic and lead about 1.5*x* and 2.5*x* higher in PM_10_ compared to the bulk tailings (Table S10 and Fig. S17†). The acidic biogeochemical environment in SGF promotes the dissolution of ferrihydrite with concomitant releases of surface complexed arsenate from the PM. Whereas reprecipitation of ferrihydrite was not expected under acidic conditions (or in an open *in vivo* system), at long protracted exposures the IVBA solution becomes supersaturated with respect to plumbojarosite, which can sequester arsenate at up to 33% of the T sites.^100^ The solid phase sequestration of lead at long exposures after 100 h was attributed to plumbojarosite and not precipitation of pyromorphite.

## 5 Conclusions

The research approach combined macroscopic studies of particle dissolution kinetics in synthetic lung and gastric biofluids with XAS of molecular speciation before, during, and after biofluid exposures. Speciation determination by XAS proved to be a powerful tool for determining molecular controls over arsenic and lead release to biofluids, and also provided key molecular-scale structural information pertaining to the host sulfate and ferric minerals in carrier dust PM.

The lead released from all PM in SLF approached an apparent maximum at ≤ 0.5 h, indicating that very short residence times in lung fluid pose important exposure risk, while the continued release of arsenic to SLF at each time step indicates longer exposures pose the greatest health risk. The greatest *in vitro* release from inhaled or ingestion fugitive dusts were expected from either metal(loid) containing soluble-efflorescent salts or PM_10_. Along with particle size and specific surface area, arsenic and lead speciation exert control over their respective kinetic released to IVBA. Speciation was a function of the oxidative weathering of the initially deposited sulfides (arsenopyrite and galena) and the metal(loid) species associated with secondary minerals (jarosite and ferrihydrite) subsequently played a key role in toxin solubility and exposure *via* release in gastric and lung fluid.

The aim of a bioassay is to provide direct data estimates of human absorption of toxic metal(loid)s, and the established *in vitro* bioassay experiments are the best tools to achieve these heath and research aims. Further, it should be recognized that IVBA is effective for prediction of arsenic and lead bioaccessibility in a wide range of mine tailings and mine impacted soils.^101^ Whereas IVBA it is not meant to model the entire complexity of the gastrointestinal tract in a simple bioassay, the limits of a closed batch system and lack of biosystem fluxes provides an incomplete representation speciation changes of ingested PM. Because the relative toxicity of particle-bound arsenic and lead depends on the bioavailability and *in vivo* speciation, which are impacted by the chemical forms ingested, *in vitro* bioassays cannot capture species dynamics in response to physiological systems (circulatory, urinary, *etc.*) where biochemical interactions can promote changes that can either increase or decrease the toxicity. Recent research implicates the gut microbiome in modulating metal(loid) toxicity, and the overall relationship between microbial activity and metal(loid) speciation, bioavailability, and toxicity is not currently understood.^102–104^ Continued *in vitro* investigation, supported by limited and targeted *in vivo* experiments, will provide valuable information for determining health risk associated with fugitive dusts.

## Author contribution

JC conceived the project and secured funding. RR and JC conceived and designed experiments. RR performed experiments and spectroscopic analysis. RR and JC wrote the manuscript.

## Conflicts of interest

The authors declare no competing financial interest.

## Supplementary Material

EM-025-D2EM00182A-s001
