# Supplementary material for: Molecular speciation controls arsenic and lead bioaccessibility in fugitive dusts from sulfidic mine tailings
Source: Environ Sci Process Impacts. 2022 Oct 3;25(2):288–303. doi: 10.1039/d2em00182a (PMC9945096; doi:10.1039/d2em00182a)
Supplement: EM-025-D2EM00182A-s001 [file EM-025-D2EM00182A-s001.pdf]

Electronic Supplementary Material (ESI) for Environmental Science: Processes & Impacts.
This journal is © The Royal Society of Chemistry 2022

# Supplementary Information

# Molecular speciation controls arsenic and lead bioaccessibility in fugitive dusts from sulfidic mine tailings

Robert A. Root[a*] and Jon Chorover[a,b]

[a] Department of Environmental Science, University of Arizona, Tucson AZ, USA.
[b] Arizona Laboratory for Emerging Contaminants, University of Arizona, Tucson AZ, USA
*Address correspondence to rroot@arizona.edu

**27 Pages: 17 Figures and 10 Tables**

| | |
|---|---|
| Figure S1 | Location Map |
| Figure S2 | $PM_{SC}$ SLF SGF Zn |
| Figure S3 | $PM_{ES}$ SLF SGF Zn |
| Figure S4 | SEM-EDS micrographs of $PM_{ES}$ |
| Figure S5 | EDS SEM- micrographs of $PM_{150}$ in SGF and SLF |
| Figure S6 | SEM micrographs of $PM_{10}$ in SLF |
| Figure S7 | XRD from $PM_{150}$ in SLF and SGF |
| Figure S8 | XRD from $PM_{10}$ in SLF and SGF |
| Figure S9 | Iron XANES pre-edge |
| Figure S10 | Iron EXAFS fits $PM_{10}$ |
| Figure S11 | Iron EXAFS fits $PM_{150}$ |
| Figure S12 | Sulfur XANES with pyrite highlight |
| Figure S13 | Sulfur LCF fits |
| Figure S14 | Arsenic EXAFS fits $PM_{10}$ |
| Figure S15 | Lead XANES and EXAFS |
| Figure S16 | $PM_{10}$ v bulk tailings elemental enrichment plot |

| | |
|---|---|
| Table S1 | Synthetic lung fluid (SLF) and synthetic gastric fluid (SGF) formulations |
| Table S2 | Iron EXAFS $PM_{10}$ fits |
| Table S3 | Iron EXAFS $PM_{150}$, jarosite, and ferrihydrite fits |
| Table S4 | Iron EXAFS $PM_{ES}$ and $PM_{SC}$ fits |
| Table S5 | Sulfur EXAFS $PM_{10}$ fits |
| Table S6 | Arsenic EXAFS $PM_{10}$ fits |
| Table S7 | Arsenic EXAFS $PM_{150}$ fits |
| Table S8 | Arsenic EXAFS $PM_{SC}$ fits |
| Table S9 | Lead LCF EXAFS fits |
| Table S10 | Elemental concentrations of $PM_{10}$ and bulk tailings |

Last updated 08/11/2022

**Figure S1**. Iron King Mine Humboldt Smelter Superfund Site map.

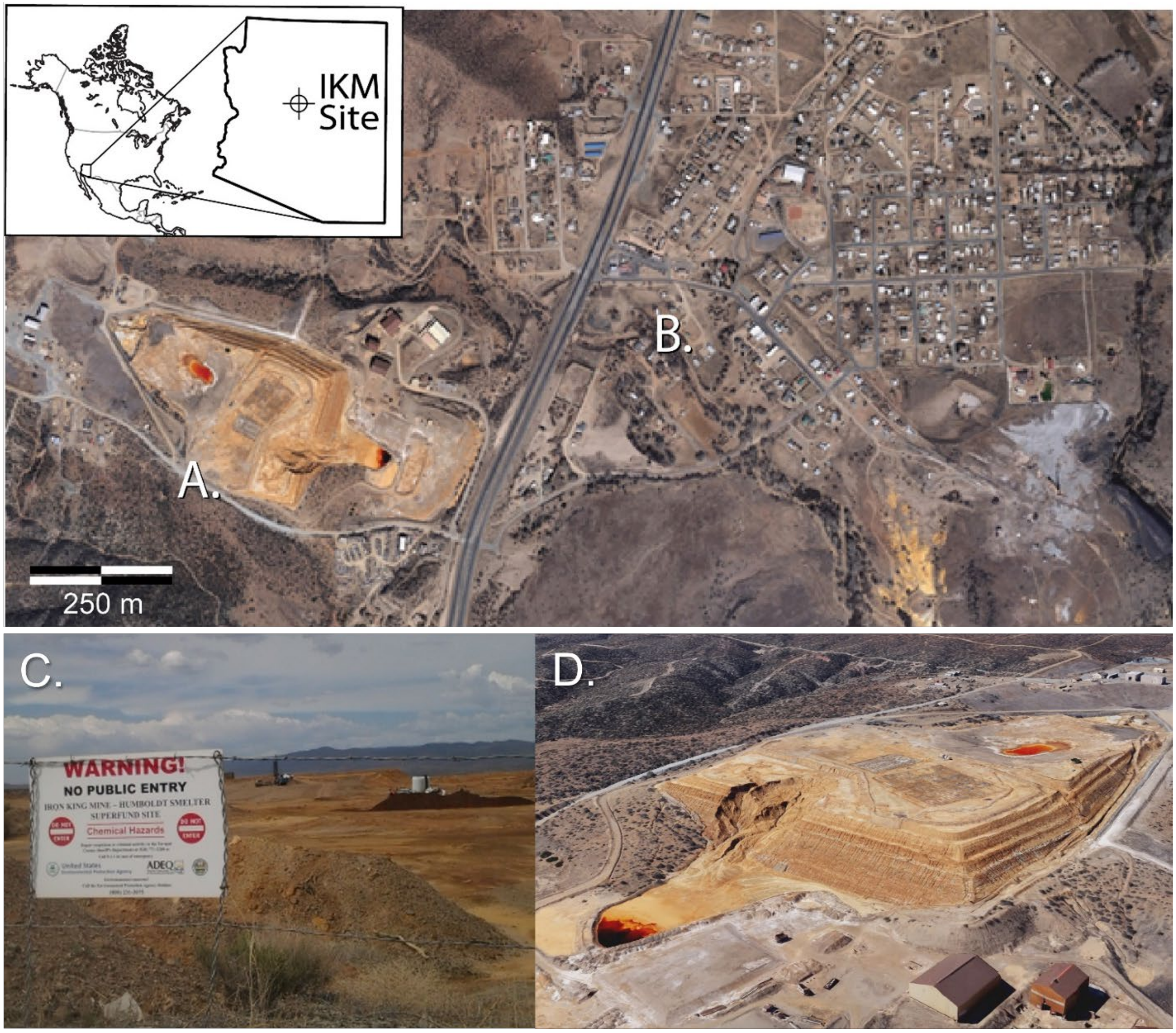


Samples were collected from the tailings pile (A). The town of Dewey-Humboldt (B) flanks the tailings. The site was designated a National Priorities Listed site in 2008 (C); contaminants of concern in the tailings (D) are arsenic and lead. Aerial photos from Google Earth®.

**Table S1.** Simulated biofluid formulations.

| Reagent | formulae | mmol $kg^{-1}$ |
|---|---|---|
| **Synthetic lung fluid (SLF)** | | |
| *Inorganic salts* | | |
| magnesium chloride | $MgCl_2 \cdot 6H_2O$ | 0.001 |
| sodium chloride | NaCl | 0.1 |
| calcium chloride | $CaCl_2 \cdot 2H_2O$ | 1.0 |
| sodium sulfate | $Na_2SO_{4CH}$ | 1.0 |
| sodium phosphate dibasic | $Na_2HPO_4$ | 1.0 |
| sodium bicarbonate | $NaHCO_3$ | 1.0 |
| *Emulgents/surfactants* | | |
| sodium tartrate dehydrate | $C_4H_4Na_2O_6 \cdot 2H_2O$ | 1.0 |
| sodium citrate dihydrate | $HOC(COONa)(CH_2COONa)_2 \cdot 2H_2O$ | 1.0 |
| *Humectant* | | |
| sodium lactate | $C_3H_5NaO_3$ | 1.0 |
| *Organic buffers* | | |
| (pH, amino acid) glycine | $NH_2CH_2COOH$ | 1.0 |
| (pe, antioxidant) sodium pyruvate | $CH_3COCOONa$ | 1.0 |
| **Synthetic gastric fluid (SGF)** | | |
| glycine | $NH_2CH_2COOH$ | 0.4 |
| hydrochloric acid | HCl | pH = 1.50 ±0.05 |

**Figure S2.** Kinetic bioaccessibility of iron, arsenic, lead, and zinc from $PM_{ES}$ in synthetic lung and gastric fluids.

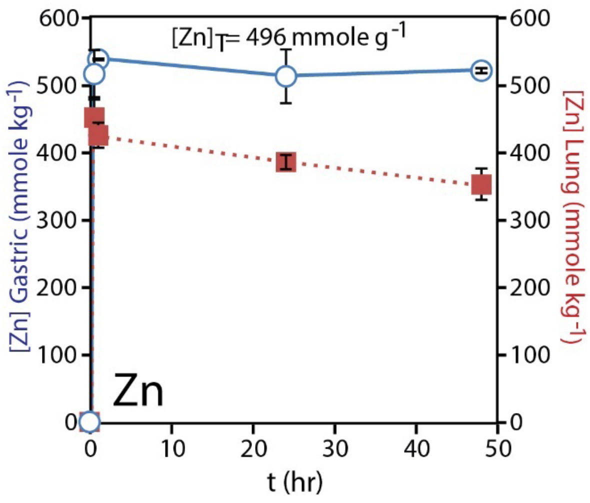


Concentration of iron, arsenic, lead, and zinc released from $PM_{ES}$ (efflorescent salts) in synthetic lung (closed red squares) and gastric (open blue circles) fluid to 48 h. Initial concentrations for each element are shown and given in Table 1.

**Figure S3.** Kinetic bioaccessibility of zinc from $PM_{SC}$ in synthetic lung and gastric fluids.

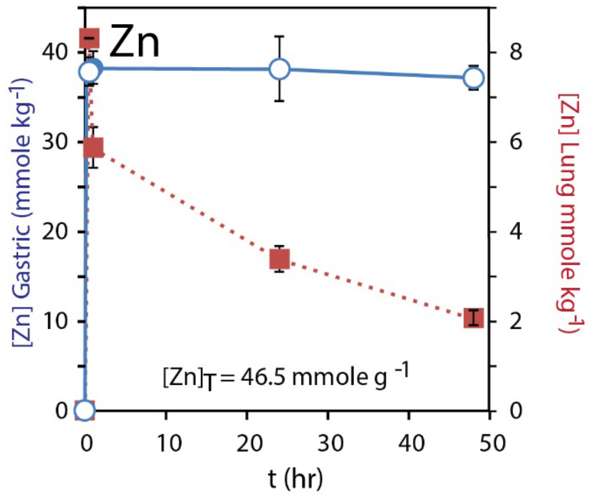


Concentration of iron, arsenic, lead, and zinc released from $PM_{sc}$ (surface crust 0-1 cm) in synthetic lung (closed red squares) and gastric (open blue circles) fluid to 48 h. Initial concentrations for each element are shown and given in Table 1.

**Figure S4.** Micrograph of unreacted $PM_{ES}$.

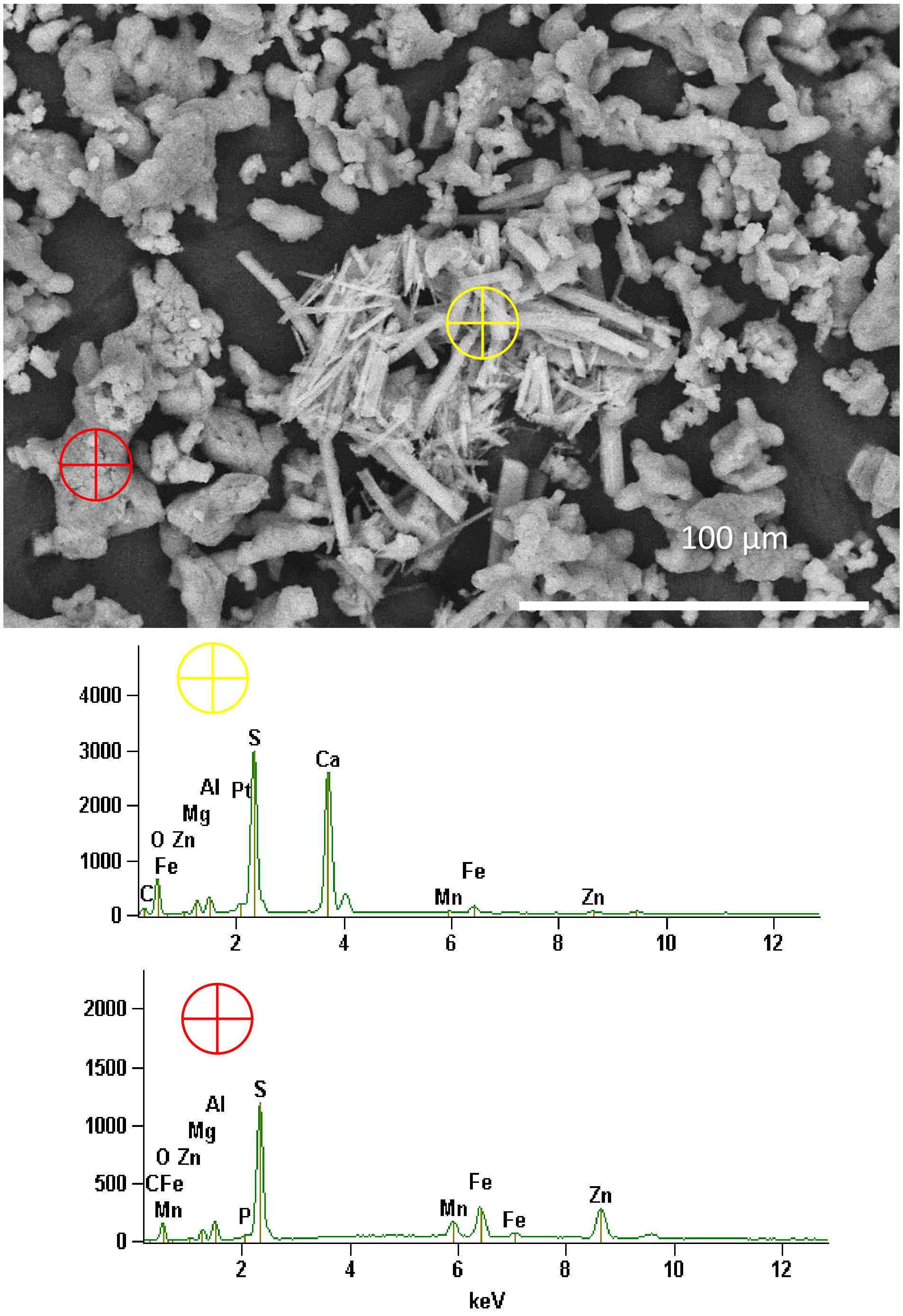


Scanning electron micrograph (SEM) and energy dispersive X-ray spectra (EDS) of efflorescent salt particulate matter ($PM_{ES}$) isolated from IKMHSS tailings before *in vitro* bioassay. EDS from the micrograph indicates Al, Ca, Mn, Fe and Zn sulfate salts. Scale bar indicates magnification.

**Figure S5.** SEM-EDS from micrographs of $PM_{150}$ in Figure 3.

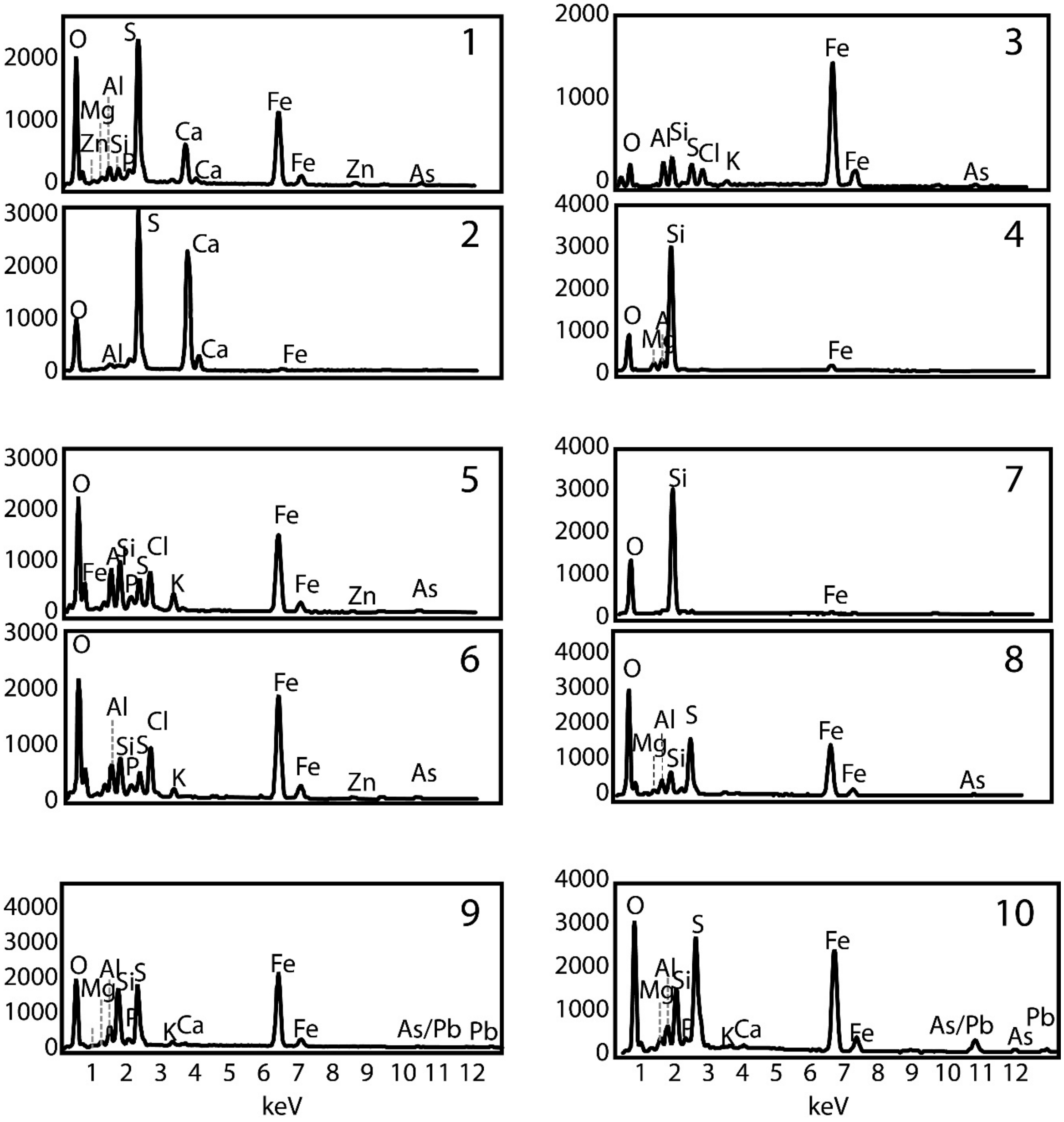


Energy dispersive X-ray spectroscopy (EDS) of <150 µm effective spherical diameter particulate matter ($PM_{150}$) isolated from IKMHSS tailings unreacted (1-2) and after *in vitro* gastric simulant bioassay for 1 hour (3-4), and 48 hours (5-6); and after *in vitro* lung simulant bioassay for 1 hour (7-8), and 48 hours (9-10). See Fig. 3 for micrographs.

**Figure S6.** Micrograph of $PM_{10}$ unreacted and after 24 h and 7 days in synthetic lung fluid.

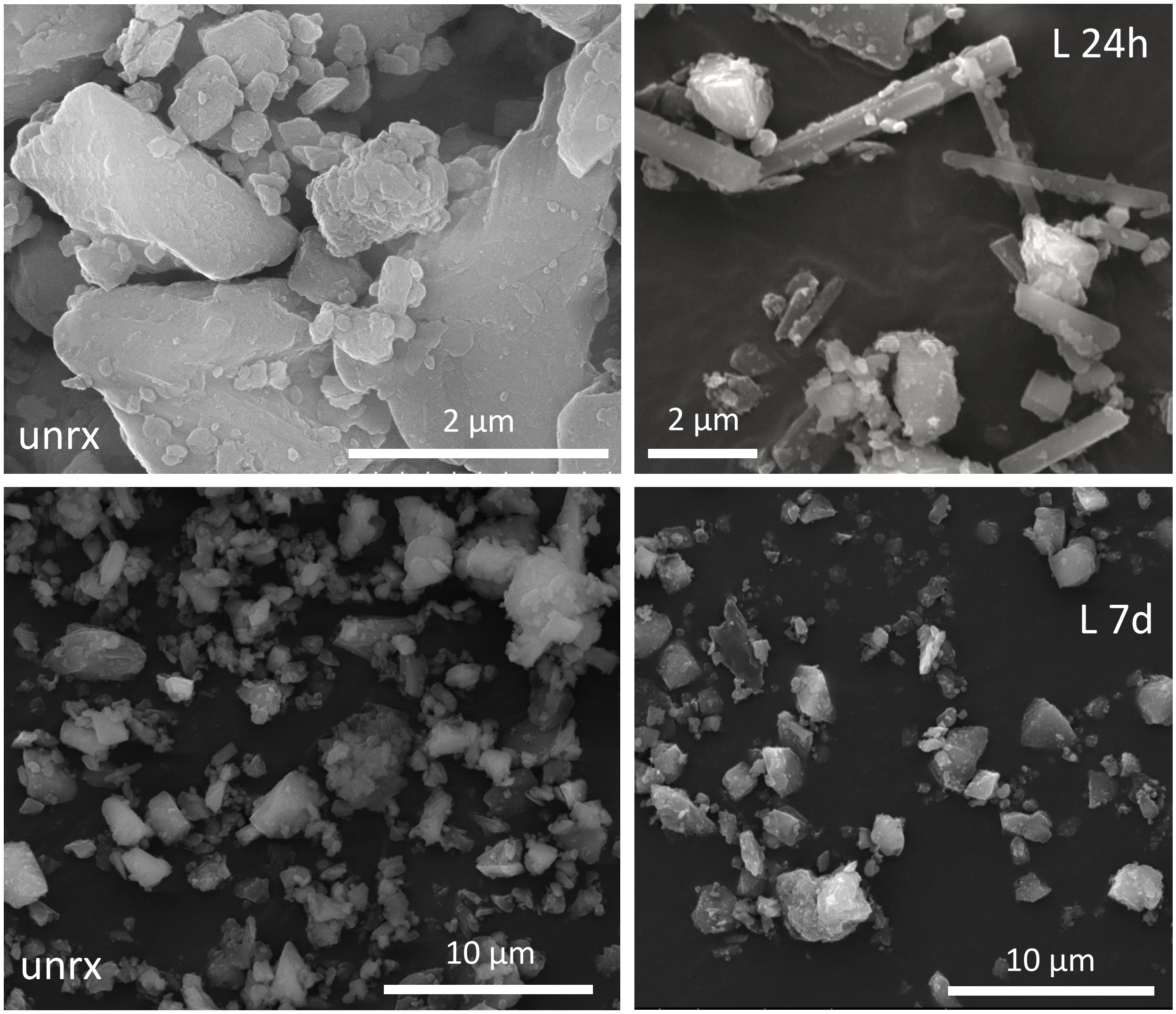


Electron micrograph of <10 µm effective spherical diameter particulate matter ($PM_{10}$) isolated from IKMHSS tailings before (left 2 panels, unrx) and after *in vitro* lung simulant bioassay for 24 hours (top right, L 24h) and 7 days (bottom right, L 7d). X-ray transparent acicular needle laths were observed 24 hours post IVBA. Scale bar indicates magnification.

**Figure S7.** X-ray diffraction of $PM_{150}$ pre and post IVBA in synthetic lung and gastric fluid.

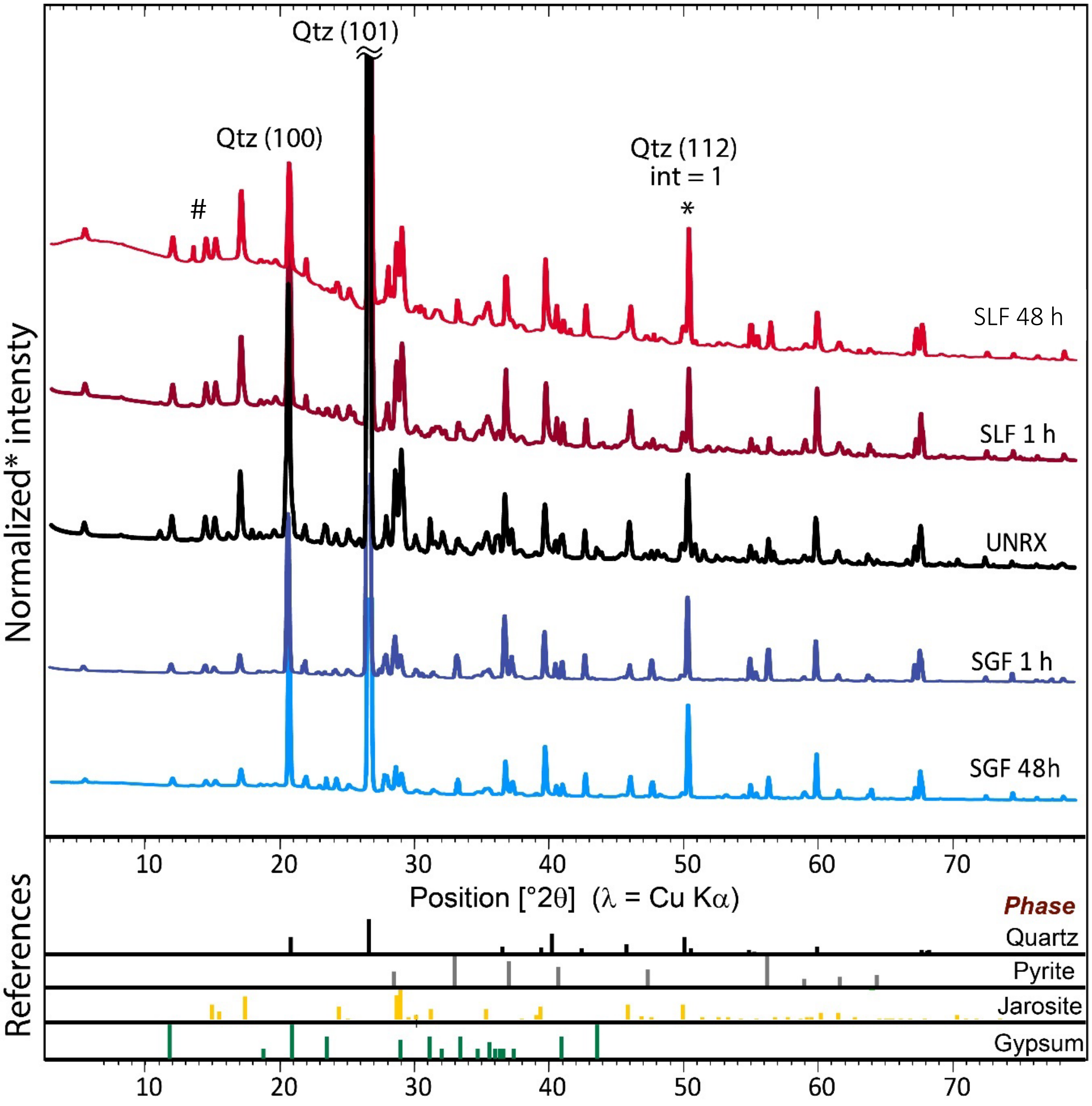


Synchrotron X-ray diffractograms collected at 0.965 Å (12.6 keV) and converted to Cu Ka at 1.5406 Å for comparison. *Diffractograms are normalized to the quartz hkl(112) peak at 1.818 Å. The black center diffractogram is the unreacted $PM_{150}$, reaction progress in SLF increases from 1 h to 48 h, shown in red, and reaction progress in SGF increases from 1 h to 48 h, shown in blue. Reference mineral peaks fitting the data are shown for quartz, pyrite, jarosite, and gypsum. # at 13.5 2θ in SLF 48h indicates the 100% line of calcium sulfate hemihydrate mineral bassanite ($CaSO_4 \cdot ½H_2O$).

**Figure S8.** X-ray diffraction of $PM_{10}$ pre and post IVBA in synthetic lung and gastric fluid.

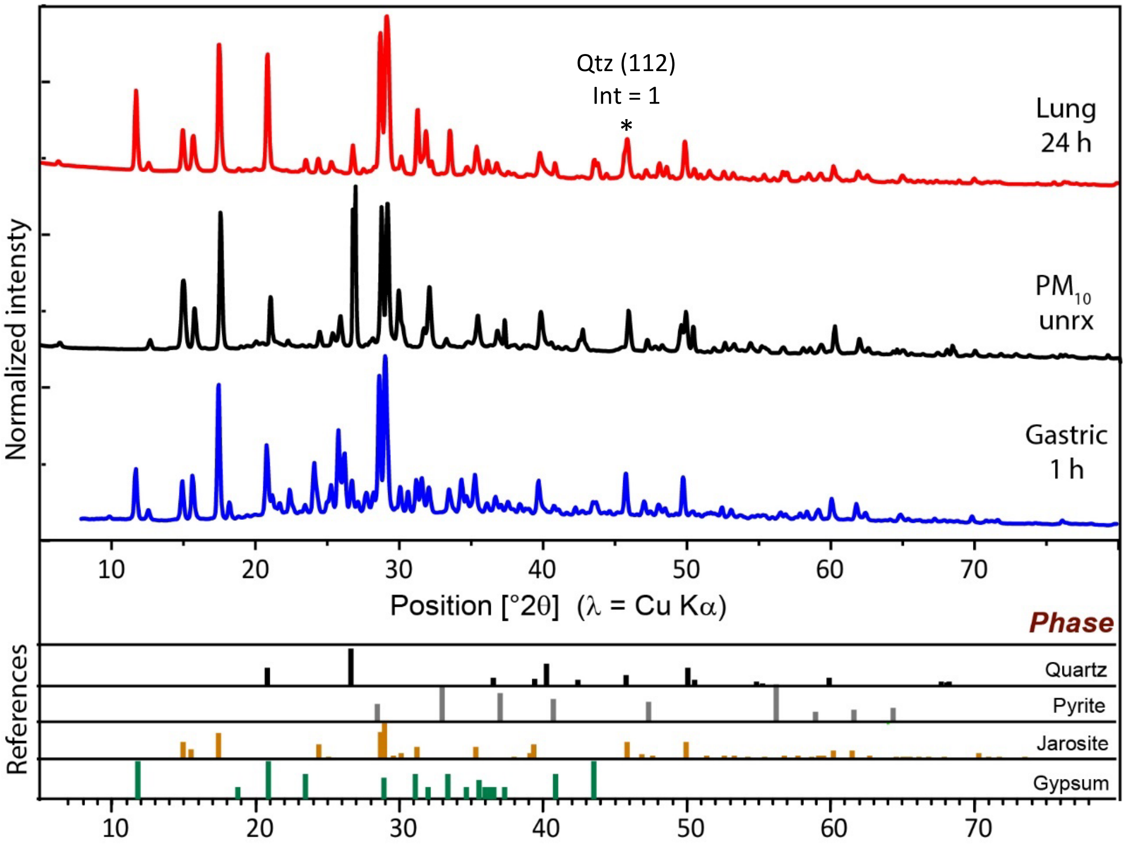


Synchrotron X-ray diffractograms collected at 0.965 Å (12.6 keV) and converted to Cu Ka at 1.5406 Å for comparison. *Diffractograms are normalized to the quartz hkl(112) peak at 1.818 Å. The black center diffractogram is the unreacted $PM_{10}$, reaction progress in SLF at 24 h is shown in red, reaction progress in SGF at 1 h is shown in blue. Reference mineral peaks fitting the data are shown for quartz, pyrite, jarosite, and gypsum.

**Figure S9.** Iron pre-edge XANES analysis of $PM_{150}$

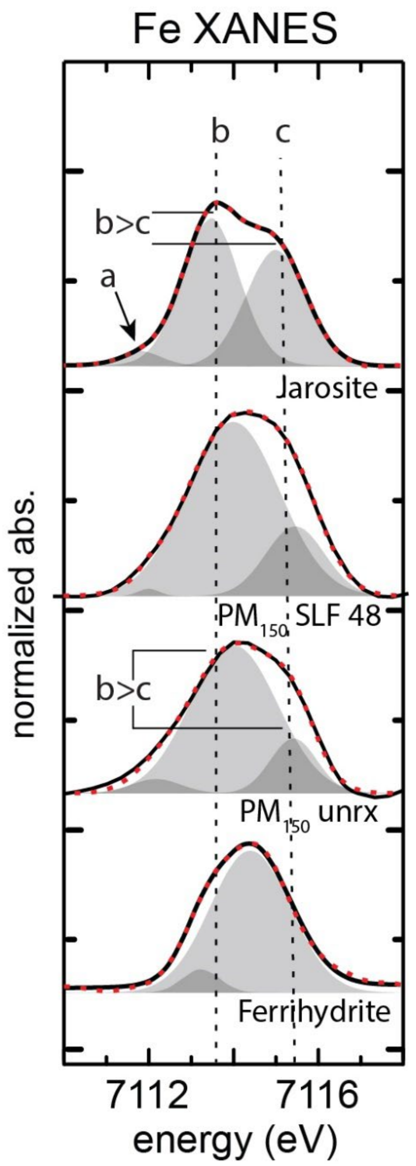


The Fe XANES pre-edge for $PM_{150}$ before and after reaction *in vitro* with synthetic lung fluid and reference jarosite and ferrihydrite. Black lines show data, red dashed lines are the fits to the data. a. pre-edge peak at 7111.9 eV present with jarosite, b. and c. are jarosite pre-edge peaks at 7113.5 eV and 7115.0 eV, where b is greater than c in jarosite; c. Overall, the pre-edge peak was observed to increase with increased ferrihydrite contribution.

**Figure S10**. Iron K-edge EXAFS of $PM_{10}$

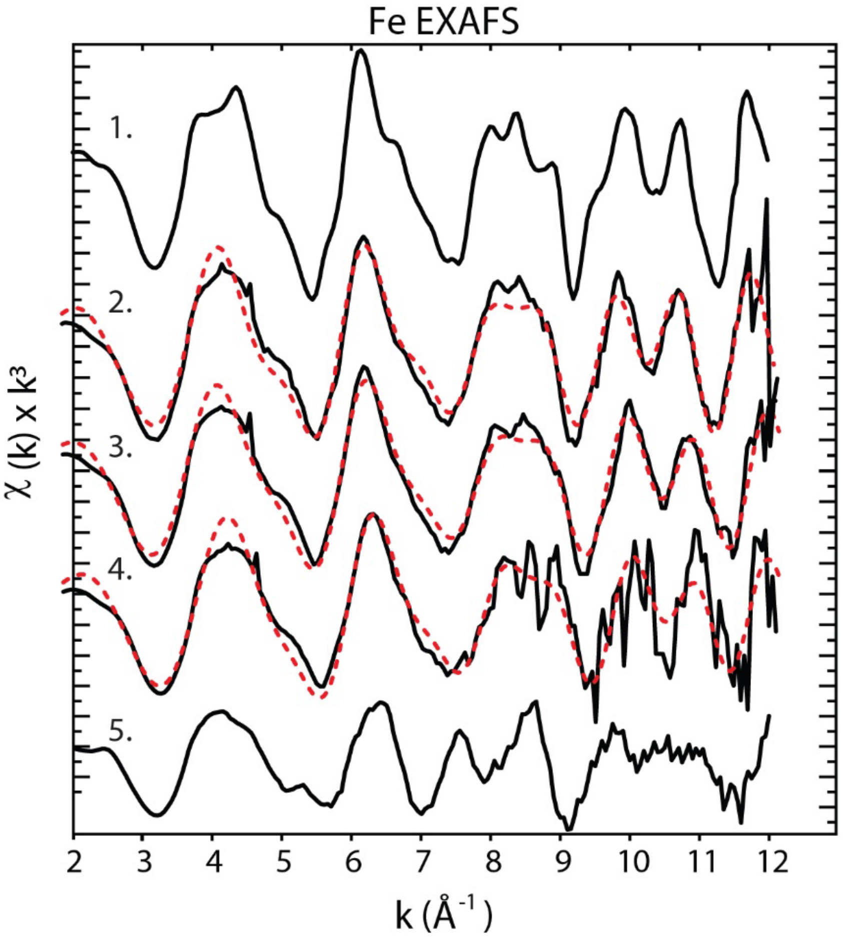


Iron K-edge EXAFS of IKMHSS $PM_{10}$ compared with reference compounds jarosite and ferrihydrite. Solid black lines are data; stippled red lines are shell by shell non-linear least-squares best fits to unfiltered $k^3$ weighted EXAFS. 1. jarosite, 2. $PM_{10}$ in SGF for 1 h, 3. $PM_{10}$ in SLF for 7 days, 4. $PM_{10}$ unreacted, 5. ferrihydrite. Fit results in Table S2.

**Figure S11.** Iron K-edge EXAFS of $PM_{150}$

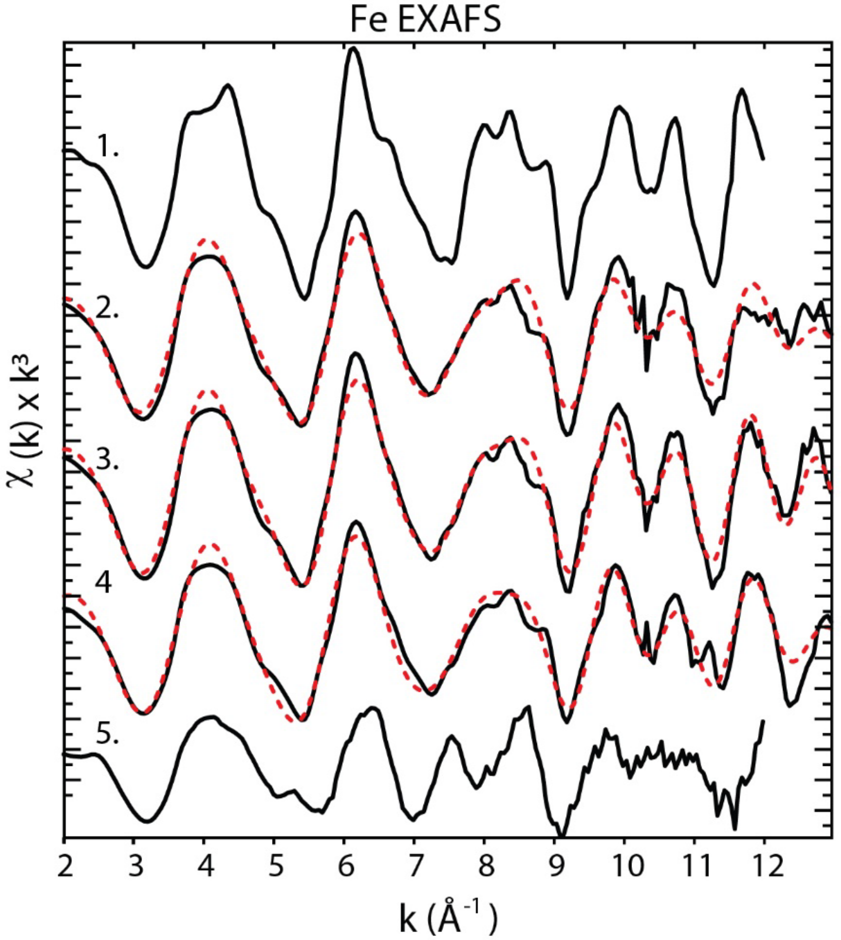


Iron K-edge EXAFS of IKMHSS $PM_{150}$ compared with reference compounds jarosite and ferrihydrite. Solid black lines are data; stippled red lines are shell by shell non-linear least-squares best fits to unfiltered $k^3$ weighted EXAFS. 1. jarosite, 2. $PM_{150}$ in SGF for 1 h, 3. $PM_{150}$ in SLF for 48h, 4. $PM_{150}$ unreacted, 5. ferrihydrite. Fit results in Table S3.

**Table S2.** Iron EXAFS fit results for $PM_{10}$

| | Jarosite Component[a] | | | | | | Ferrihydrite Component[b] | | | | | |
|---|---|---|---|---|---|---|---|---|---|---|---|---|
| sample | Atom | N | R (Å) | $\sigma^2$ ($Å^2$) | var[c] | $N_{idp}$[c] | Atom | N | R (Å) | $\sigma^2$ ($Å^2$) | $\Delta E_0$ (eV) | $\chi^2$ |
| *PM10* | | | | | | | | | | | | |
| unrx | O | 1.9 | 1.94 | 0.003 | 11 | 22 | Fe | 1.5 | 3.08 | 0.003 | 1.1 | 2.43 |
| | O | 4.1 | 2.01 | / | | | Fe | 3.1 | 3.35 | / | | |
| | S | 1.6 | 3.27 | / | | | | | | | | |
| | Fe | 3.2 | 3.61 | / | | | | | | | | |
| L-1h | O | 1.4 | 1.95 | 0.004 | 12 | 22 | Fe | 0.7 | 3.08 | 0.004 | 0.80 | 0.80 |
| | O | 4.6 | 2.00 | / | | | Fe | 1.4 | 3.35 | / | | |
| | S | 1.4 | 3.25 | / | | | | | | | | |
| | Fe | 2.9 | 3.63 | / | | | | | | | | |
| L-48h | O | 3.1 | 1.97 | 0.004 | 11 | 22 | Fe | 0.9 | 3.06 | 0.004 | 0.54 | 0.74 |
| | O | 2.9 | 2.02 | / | | | Fe | 1.7 | 3.35 | / | | |
| | S | 1.5 | 3.25 | / | | | | | | | | |
| | Fe | 3.0 | 3.64 | / | | | | | | | | |
| L-7d | O | 1.1 | 1.95 | 0.004 | 11 | 22 | Fe | 1.1 | 3.08 | 0.004 | 0.63 | 0.77 |
| | O | 4.9 | 2.01 | / | | | Fe | 2.1 | 3.37 | / | | |
| | S | 1.4 | 3.31 | / | | | | | | | | |
| | Fe | 2.9 | 3.62 | / | | | | | | | | |
| G-1h[d] | O | 2.5 | 1.96 | 0.003 | 12 | 22 | Fe | 0.8 | 3.08 | 0.003 | 0.88 | 0.89 |
| | O | 3.5 | 2.02 | / | | | Fe | 1.6 | 3.37 | / | | |
| | S | 1.5 | 3.29 | / | | | | | | | | |
| | Fe | 2.9 | 3.63 | / | | | | | | | | |
| G-48[d] | O | 1.9 | 1.94 | 0.003 | 11 | 22 | Fe | 0.6 | 3.07 | 0.003 | 1.0 | 0.83 |
| | O | 4.1 | 2.03 | / | | | Fe | 1.1 | 3.36 | / | | |
| | S | 1.0 | 3.29 | / | | | | | | | | |
| | Fe | 2.0 | 3.63 | / | | | | | | | | |

Fe EXAFS fit (k=2-12) constrained by jarosite and ferrihydrite structures. The amplitude reduction factor was fixed at $S^0 = 0.7$. Due to the covariance of coordination numbers (N) and the Debye-Waller term ($\sigma^2$), $\sigma^2$ were fixed based on best agreement to reference fits from crystallographic values from (O'Day 2004; Savage 2005), interatomic distances (R) were iteratively varied, $\sigma^2$ was a linked parameter anchored to the Fe-O backscattering contribution and held constant. [a]Jarosite component: [b]Ferrihydrite component: [c] Degrees of freedom in the signal $N_{idp}= 2\Delta k\Delta R/\pi$, k= 2 - 12 ($\Delta k = 10$) and R= 1 - 4.5 ($\Delta R = 3.5$), the number of variables must not exceed the degrees of freedom, $N_{idp}$ was 22, fits were constrained to 12 or fewer variables; / parameter linked in fit to the parameter directly above. Estimated errors for R ± 0.02 Å, N or σ2 ± 30% based on empirical fits to known reference compounds were for atoms beyond the first shell (see O'Day et al., 2004).

**Table S3.** Iron EXAFS fit results for $PM_{150}$ and reference jarosite and ferrihydrite

| Jarosite Component[a] | | | | | | | Ferrihydrite Component[b] | | | | | |
|---|---|---|---|---|---|---|---|---|---|---|---|---|
| Sample | Atom | N | R (Å) | $\sigma^2$ ($Å^2$) | var[c] | $N_{idp}$[c] | Atom | N | R (Å) | $\sigma^2$ ($Å^2$) | $\Delta E_0$ (eV) | $\chi^2$ |
| *$PM_{150}$* | | | | | | | | | | | | |
| Unrx | O | 6.0 | 2.00 | 0.006 | 11 | 22 | Fe | 0.4 | 3.04 | 0.003 | 0.51 | 0.595 |
| | S | 1.7 | 3.25 | 0.003 | | | Fe | 1.2 | 3.38 | / | | |
| | Fe | 2.3 | 3.62 | / | | | | | | | | |
| G-1h | O | 6.0 | 1.98 | 0.006 | 11 | 22 | Fe | 0.5 | 3.02 | 0.003 | -2.1 | 0.652 |
| | S | 1.7 | 3.24 | 0.003 | | | Fe | 0.8 | 3.34 | / | | |
| | Fe | 1.0 | 3.63 | / | | | | | | | | |
| L 48h | O | 6.0 | 1.97 | 0.006 | 11 | 22 | Fe | 0.7 | 3.06 | 0.003 | 1.6 | 0.391 |
| | S | 1.4 | 3.25 | 0.003 | | | Fe | 0.9 | 3.32 | / | | |
| | Fe | 1.2 | 3.64 | / | | | | | | | | |
| *Jarosite[a]* | | | | | | | | | | | | |
| | O | 6.0 | 1.99 | 0.005 | 7 | 22 | - | - | - | - | 2.2 | 1.578 |
| | S | 2.0 | 3.26 | / | | | | | | | | |
| | Fe | 4.0 | 3.66 | / | | | | | | | | |
| *Ferrihydrite[b]* | | | | | | | | | | | | |
| | O | 6.0 | 2.01 | 0.007 | 5 | 22 | Fe | 0.5 | 3.05 | 0.006 | 2.1 | 0.758 |
| | | | | | | | Fe | 0.8 | 3.43 | 0.007 | | |

EXAFS fit (k=2-12) constrained as above in Table S2. $S^0$ = 0.7, $\sigma^2$ fixed based on best agreement to reference fits (O'Day et al., 2004; Savage et al., 2005), R values were iteratively varied, $\sigma^2$ was as a linked parameter, / parameter linked in fit to the parameter directly above. [a]Jarosite and [b]Ferrihydrite components described in the text. [c] $N_{ipd}= 2\Delta k\Delta R/\pi$, k= 2 - 12 ($\Delta k$ = 10) and R= 1 - 4.5 ($\Delta R$ = 3.5), $N_{ipd}$ was 22, fits were constrained to 12 or fewer variables. . Estimated errors for R ± 0.02 Å, N or σ2 ± 30% based on empirical fits to known reference compounds were for atoms beyond the first shell (see O'Day et al., 2004).

**Table S4.** Iron EXAFS fit results for $PM_{ES}$ and $PM_{SC}$

| | Jarosite Component[a] | | | | | | Ferrihydrite Component[b] | | | | | |
|---|---|---|---|---|---|---|---|---|---|---|---|---|
| sample | Atom | N | R (Å) | $\sigma^2$ ($Å^2$) | var | $N_{idp}$ | Atom | N | R (Å) | $\sigma^2$ ($Å^2$) | $\Delta E_0$ (eV) | $\chi^2$ |
| *$PM_{ES}$* | | | | | | | | | | | | |
| unrx | O | 6.0 | 2.00 | 0.005 | 11 | 22 | Fe | 0.4 | 3.26 | 0.005 | -1.6 | 0.701 |
| | S | 1.5 | 3.27 | / | | | Fe | 1.6 | 3.40 | / | 1.86 | |
| | Fe | 2.2 | 3.62 | / | | | | | | | | |
| L 48h | O | 6.0 | 1.98 | 0.005 | 11 | 22 | Fe | 1.0 | 3.04 | 0.005 | -0.6 | 0.787 |
| | S | 0.5 | 3.12 | / | | | Fe | 1.1 | 3.50 | / | | |
| | Fe | 2.0 | 3.67 | / | | | | | | | | |
| *$PM_{SC}$* | | | | | | | | | | | | |
| unrx | O | 6.0 | 2.00 | 0.006 | 11 | 22 | Fe | 0.4 | 3.05 | 0.003 | -0.67 | 0.517 |
| | S | 1.5 | 3.25 | 0.003 | | | Fe | 1.1 | 3.39 | / | | |
| | Fe | 2.3 | 3.63 | / | | | | | | | | |
| G-1h | O | 6.0 | 1.99 | 0.006 | 11 | 22 | Fe | 0.6 | 3.03 | 0.003 | -0.67 | 0.622 |
| | S | 1.1 | 3.25 | 0.003 | | | Fe | 0.6 | 3.37 | / | | |
| | Fe | 1.6 | 3.64 | / | | | | | | | | |

EXAFS fit (k=2-12) constrained as above in Table S2 and S3. $S^0 = 0.7$, $\sigma^2$ fixed based on best agreement to reference fits (O'Day 2004; Savage 2005), R values were iteratively varied, $\sigma^2$ was as a linked parameter, / parameter linked in fit to the parameter directly above.. [a]Jarosite and [b]Ferrihydrite components described in the text. [c] $N_{ipd}= 2\Delta k\Delta R/\pi$, k= 2 - 12 ($\Delta k = 10$) and R= 1 - 4.5 ($\Delta R = 3.5$), $N_{ipd}$ was 22, fits were constrained to 12 or fewer variables. . Estimated errors for R ± 0.02 Å, N or σ2 ± 30% based on empirical fits to known reference compounds were for atoms beyond the first shell (see O'Day et al., 2004).

**Figure S12** . Sulfur K-edge XANES

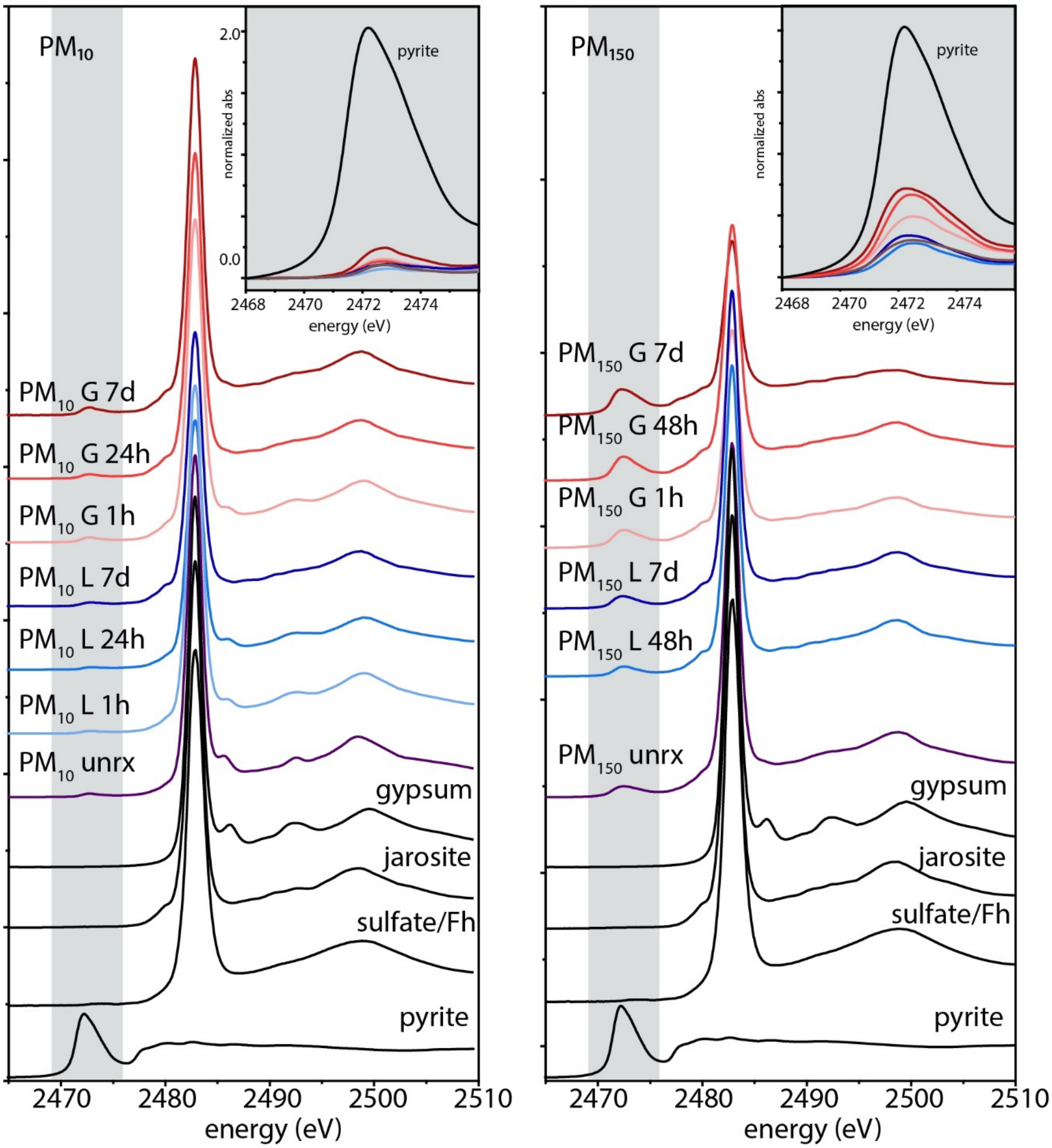


Sulfur K-edge XANES of IKMHSS $PM_{10}$ (left) and $PM_{150}$ (right) with reference minerals used in least squares linear combination fitting. The shaded vertical region highlights the pyrite contribution, and the inset shows the detailed pyrite contribution from each PM. The spectra show the relative growth of the pyrite contribution with reaction time in SLF and SGF due to dissolution of the sulfate species. Fits shown in Fig. S13 and Table S5.

**Figure S13.** Linear combination fits of sulfur K-edge XANES

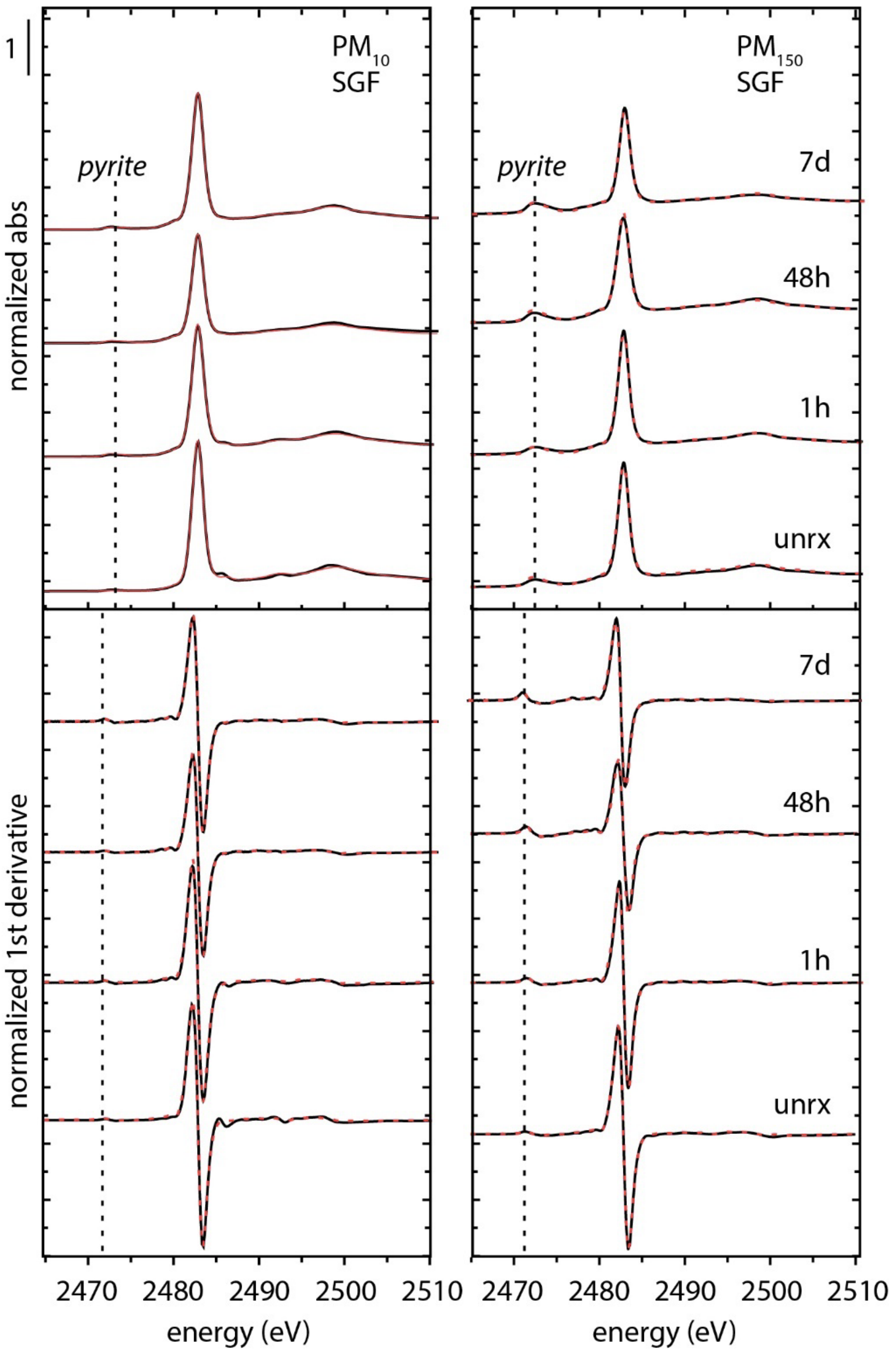


Least-squares linear combination fits of sulfur K-edge XANES of IKMHSS $PM_{10}$ (left) and $PM_{150}$ (right) fit to the normalize post edge (top) and first derivative (bottom). Solid black lines are data; stippled red lines are least-squares best fits. Model compounds are shown in Fig, S12. The vertical line points out the contribution from pyrite. (fit results in Table S5). The spectra show that sulfate species (gypsum, jarosite and sulfate associated ferrihydrite) are dissolved relative to pyrite in the pH 1.5 gastric fluid.

**Table S5.** SLF and SGF linear combination fits of S XANES for $PM_{10}$ and $PM_{150}$ tailings.

| $PM_{10}$ | Gyp[a] | Jar[b] | Pyt[c] | Fh-$SO_4$[d] | Σ[e] | *R*-factor[f] | $\chi^2$[g] |
|---|---|---|---|---|---|---|---|
| Unrx | 0.491 | 0.323 | 0.031 | 0.126 | 0.971 | 0.0017 | 0.0068 |
| L1h | 0.432 | 0.337 | 0.034 | 0.198 | 1.001 | 0.0005 | 0.0023 |
| L24 | 0.440 | 0.293 | 0.045 | 0.110 | 0.888 | 0.0002 | 0.0009 |
| L7d | | 0.533 | 0.096 | 0.218 | 0.847 | 0.0008 | 0.0027 |
| G1h | 0.207 | 0.284 | 0.124 | 0.378 | 1.001 | 0.0005 | 0.0023 |
| G24 | 0.071 | 0.100 | 0.094 | 0.130 | 0.888 | 0.0002 | 0.0009 |
| **$PM_{150}$** | | | | | | | |
| Unrx | 0.128 | 0.599 | 0.181 | 0.121 | 1.029 | 0.0004 | 0.0011 |
| L7 | | 0.815 | 0.185 | | 0.977 | 0.0002 | 0.0007 |
| G1h | | 0.680 | 0.255 | | 0.997 | 0.0044 | 0.0076 |
| G24 | | 0.850 | 0.127 | | 0.948 | 0.0030 | 0.0056 |
| G48 | | 0.611 | 0.386 | | 1.000 | 0.0040 | 0.0146 |

Fractional contribution of reference mineral to $PM_{10}$ and $PM_{150}$. Reference minerals [a]gypsum, [b]jarosite, [c]pyrite, and [d]sulfate sorbed to ferrihydrite; [e] sum of the fractional fit components not normalized to unity, [f] reduced *R*-factor, and [g] $\chi^2$ are given as goodness-of-fit parameters; PM samples are unrx = unreacted, reacted in SLF for 1 hour, L1h; 24 hours, L24; 7 days L7d; and in SGLF for 1 hour, G1; 24 hours,GL24; 48 hours, G48. Fits shown in Fig. S13.

**Figure S14.** $PM_{10}$ As EXAFS Fourier transform

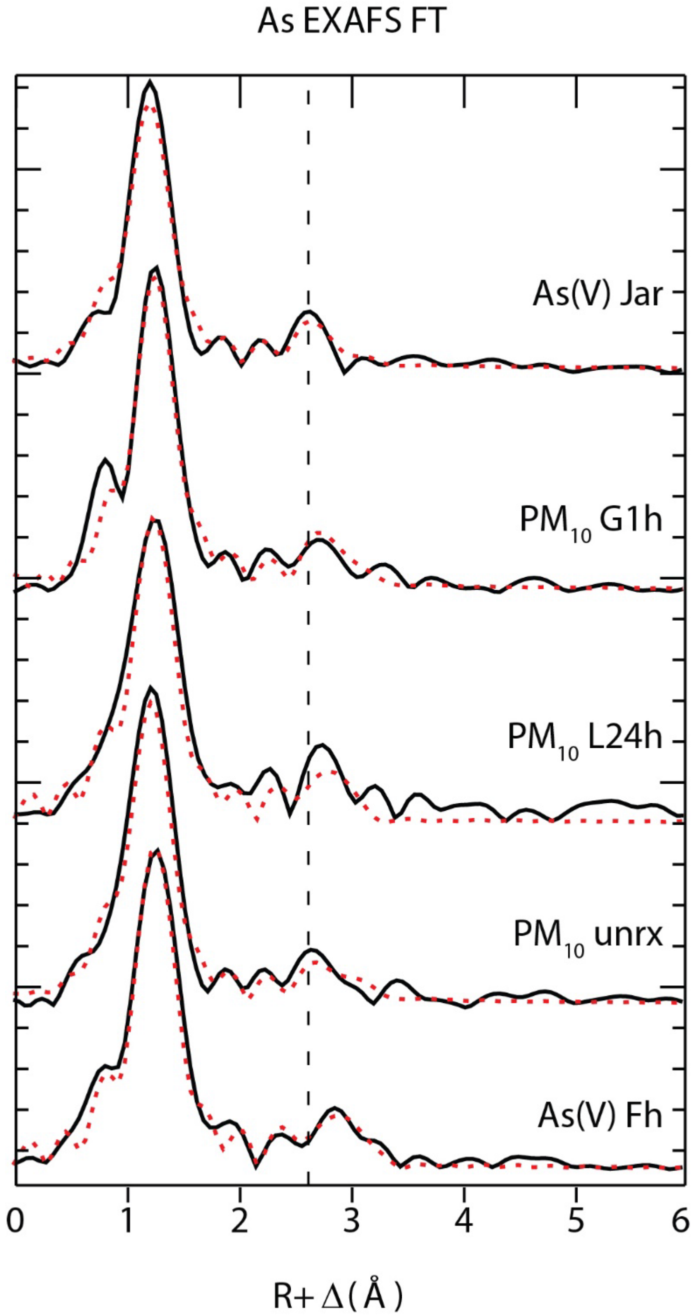


Arsenic K-edge Fourier transformed EXAFS of IKMHSS $PM_{10}$ before and after *in vitro* bioassay with synthetic gastric fluid (SGF) or synthetic lung fluid (SLF) compared with reference jarosite and ferrihydrite. Solid black lines are data; stippled red lines are shell by shell non-linear least-squares best fits; unfiltered $k^3$ and uncorrected for phase shift FT. Model compounds are shown. The vertical line points out second shell contribution from iron backscatter atoms of ferrihydrite and jarosite and highlight the structural features corresponding to the calculated coordination and distance, explained in the text. (fit results in Table S6).

**Table S6**. Arsenic EXAFS fit results for $PM_{10}$

| fit parameters | | | | | fit error analysis | | | |
|---|---|---|---|---|---|---|---|---|
| Sample | Atom | N | R (Å) | $\sigma^2$ (Å$^2$) | N | R (Å) | $\Delta E_0$ (eV) | $\chi^2$ |
| *$PM_{10}$* | | | | | | | | |
| unrx | O | 4.0 | 1.68 | 0.0030 | ±0.07 | ±0.002 | -3.1 | 1.16 |
| | MS | 12.0 | 3.05 | 0.0045/ | / | / | | |
| | Fe | 2.2 | 3.32 | 0.008 | ±0.23 | ±0.007 | | |
| G-1h | O | 4.3 | 1.69 | 0.0020 | ±0.09 | ±0.002 | -5.4 | 2.12 |
| | MS | 12.9 | 3.08 | 0.0030/ | / | / | | |
| | Fe | 2.1 | 3.30 | 0.008 | ±0.28 | ±0.009 | | |
| G-24 | O | 4.2 | 1.68 | 0.0025 | ±0.09 | ±0.002 | -6.3 | 1.89 |
| | MS | 12.5 | 3.07 | 0.0038 | / | / | | |
| | Fe | 1.0 | 3.29 | 0.008 | ±0.3 | ±0.009 | | |
| L-24h | O | 4.0 | 1.69 | 0.0024 | ±0.3 | ±0.0002 | -6.1 | 2.12 |
| | MS | 12 | 3.11 | 0.0036 | / | / | | |
| | Fe | 1.9 | 3.31 | 0.008 | ±0.3 | ±0.01 | | |
| L-48h | O | 4.3 | 1.69 | 0.002 | ±0.2 | ±0.010 | -5.9 | 1.50 |
| | MS | 13.0 | 3.08 | 0.003 | / | / | | |
| | Fe | 1.2 | 3.34 | 0.008 | | | | |
| L-7d | O | 4.0 | 1.69 | 0.0024 | ±0.3 | ±0.0002 | -6.4 | 2.18 |
| | MS | 12.0 | 3.08 | 0.0036/ | / | / | | |
| | Fe | 1.7 | 3.30 | 0.008 | ±0.2 | ±0.009 | | |
| Jarosite | O | 4.16 | 1.66 | 0.0030 | ±0.09 | ±0.029 | -5.5 | 1.88 |
| | MS | 12.4 | 3.05 | 0.0045 | / | / | | |
| | Fe | 3.0 | 3.27 | 0.0104 | ±0.01 | ±0.001 | | |
| Fh low pH | O | 4.0 | 1.70 | 0.002 | ±0.002 | ±0.0001 | -3.5 | 0.95 |
| | MS | 12.0 | 3.10 | 0.003 | / | / | | |
| | Fe | 2.2 | 3.27 | 0.008 | ±0.01 | ±0.001 | | |

Due to the covariance of coordination numbers (N) and the Debye-Waller term ($\sigma^2$), N were fixed based on crystallographic values of arsenate tetrahedra (O'Day 2004; Savage 2005), interatomic distances (R) was iteratively varied and the $\sigma^2$ was allowed to vary as a linked parameter anchored to the As-Fe backscattering contribution and held constant; / parameter linked in fit to the parameter directly above. Degrees of freedom in the signal $N_{idp}= 2\Delta k\Delta R/\pi$, k= 1 - 12 ($\Delta$k= 11) and R= 1 - 4.5 ($\Delta$R = 3.5); the number of variables must not exceed the degrees of freedom, $N_{idp}$ was 24, fits were constrained to 6 or fewer variables. Estimated errors for R ± 0.02 Å, N or σ2 ± 30% based on empirical fits to known reference compounds were for atoms beyond the first shell (see O'Day et al., 2004).Spectral fits shown in Fig. S14.

**Table S7.** Arsenic EXAFS fit results for $PM_{150}$

| fit parameters | | | | | fit error analysis | | | |
|---|---|---|---|---|---|---|---|---|
| sample | Atom | N | R (Å) | $\sigma^2$ ($Å^2$) | N | R (Å) | $\Delta E_0$ (eV) | $\chi^2$ |
| *$PM_{150}$* | | | | | | | | |
| unrx | O | 4.0 | 1.68 | 0.003 | ±0.003 | ±0.0002 | -7.5 | 2.05 |
| | MS | 12.0 | 3.06 | 0.004/ | / | / | | |
| | Fe | 1.5 | 3.28 | 0.008 | ±0.28 | ±0.012 | | |
| G-1h | O | 4.0 | 1.68 | 0.003 | ±0.002 | ±0.0002 | -6.4 | 1.56 |
| | MS | 12.0 | 3.13 | 0.004/ | / | / | | |
| | Fe | 2.2 | 3.27 | 0.008 | ±0.24 | ±0.008 | | |
| G-24 | O | 4.0 | 1.68 | 0.003 | ±0.003 | ±0.0002 | -7.0 | 1.97 |
| | MS | 12.0 | 3.07 | 0.004/ | / | / | | |
| | Fe | 2.2 | 3.28 | 0.008 | ±0.28 | ±0.009 | | |
| L-48h | O | 4.0 | 1.68 | 0.003 | ±0.003 | ±0.0002 | -6.7 | 2.09 |
| | MS | 12 | 3.12 | 0.004/ | | | | |
| | Fe | 1.6 | 3.32 | 0.008 | ±0.29 | ±0.012 | | |
| L-7d | O | 4.0 | 1.69 | 0.003 | ±0.3 | ±0.0002 | | |
| | MS | 12.0 | 3.08 | 0.004/ | | | | |
| | Fe | 1.7 | 3.30 | 0.008 | ±0.2 | ±0.009 | | |
| Jarosite | O | 4.16 | 1.66 | 0.003 | ±0.09 | ±0.029 | -5.5 | 1.88 |
| | MS | 12.4 | 3.05 | 0.004/ | / | / | | |
| | Fe | 3.0 | 3.27 | 0.0104 | ±0.01 | ±0.001 | | |
| Fh | O | 4.0 | 1.70 | 0.002 | ±0.002 | ±0.0001 | -3.5 | 0.95 |
| low pH | MS | 12.0 | 3.10 | 0.003/ | / | / | | |
| | Fe | 2.2 | 3.27 | 0.008 | ±0.01 | ±0.001 | | |

Due to the covariance of coordination numbers (N) and the Debye-Waller term ($\sigma^2$), N were fixed based on crystallographic values of arsenate tetrahedra (O'Day 2004; Savage 2005), interatomic distances (R) was iteratively varied and the $\sigma^2$ was allowed to vary as a linked parameter anchored to the As-Fe backscattering contribution and held constant. / parameter linked in fit to the parameter directly above. Degrees of freedom in the signal $N_{idp}= 2\Delta k\Delta R/\pi$, k= 1 - 12 ($\Delta k$= 11) and R= 1 - 4.5 ($\Delta R$ = 3.5); the number of variables must not exceed the degrees of freedom, $N_{idp}$ was 24, fits were constrained to 6 or fewer variables. Estimated errors for R ± 0.02 Å, N or σ2 ± 30% based on empirical fits to known reference compounds were for atoms beyond the first shell (see O'Day et al., 2004).

**Table S8.** Arsenic EXAFS fit results for $PM_{SC}$ surface crust and reference ferrihydrite.

| fit parameters | | | | | fit error analysis | | | |
|---|---|---|---|---|---|---|---|---|
| sample | Atom | N | R (Å) | $\sigma^2$ (Å$^2$) | N | R (Å) | $\Delta E_0$ (eV) | $\chi^2$ |
| *Surface crust* | | | | | | | | |
| unrx | O | 4.0 | 1.68 | 0.003 | ±0.003 | ±0.0002 | -6.4 | 1.92 |
| | MS | 12.0 | 3.06 | 0.004/ | / | / | | |
| | Fe | 1.2 | 3.28 | 0.008 | ±0.22 | ±0.012 | | |
| G-1h | O | 4.0 | 1.68 | 0.003 | ±0.002 | ±0.0002 | -8.5 | 1.75 |
| | MS | 12.0 | 3.07 | 0.004/ | / | / | | |
| | Fe | 1.6 | 3.27 | 0.008 | ±0.19 | ±0.011 | | |
| G-24 | O | 4.0 | 1.68 | 0.003 | ±0.003 | ±0.0002 | -7.0 | 1.97 |
| | MS | 12.0 | 3.07 | 0.004/ | / | / | | |
| | Fe | 2.2 | 3.28 | 0.008 | ±0.28 | ±0.009 | | |

Due to the covariance of coordination numbers (N) and the Debye-Waller term ($\sigma^2$), N were fixed based on crystallographic values of arsenate tetrahedra (O'Day 2004; Savage 2005), interatomic distances (R) was iteratively varied and the $\sigma^2$ was allowed to vary as a linked parameter anchored to the As-Fe backscattering contribution and held constant; / parameter linked in fit to the parameter directly above. [c] Degrees of freedom in the signal $N_{idp}= 2\Delta k\Delta R/\pi$, k= 1 - 12 ($\Delta$k= 11) and R= 1 - 4.5 ($\Delta$R = 3.5); the number of variables must not exceed the degrees of freedom, $N_{idp}$ was 24, fits were constrained to 6 or fewer variables. Estimated errors for R ± 0.02 Å, N or σ2 ± 30% based on empirical fits to known reference compounds were for atoms beyond the first shell (see (1)).

**Figure S15.** Lead $L_{III}$ XANES and EXAFS of Iron King mine tailings.

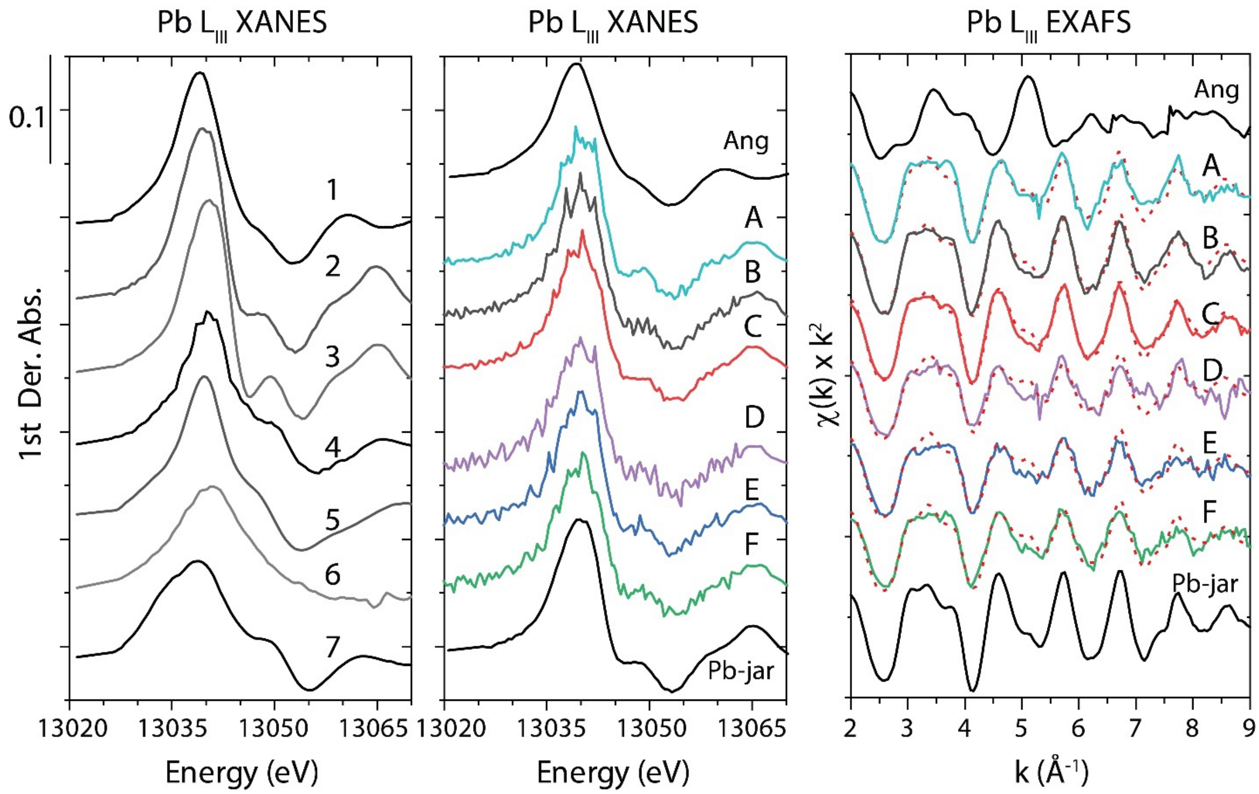


Unreacted and IVBA reacted $PM_{10}$ and $PM_{150}$. Left panel: lead XANES references: 1. anglesite, 2. plumbojarosite, 3. beaverite, 4. pyromorphite, 5. cerussite, 6. lead adsorbed to ferrihydrite, 7. galena. Center panel: lead XANES of $PM_{10}$ and $PM_{150}$ A. $PM_{10}$ SGF 1 h, B. $PM_{10}$ SGF 7 day, C. $PM_{10}$ unreacted, D. $PM_{150}$ SGF 1 h, E. $PM_{150}$ SGF 7 day, F. $PM_{150}$ unreacted, reference anglesite and plumbojarosite shown. Right panel: lead EXAFS fit of $PM_{10}$ and $PM_{150}$ A. $PM_{10}$ SGF 1 h, B. $PM_{10}$ SGF 7 day, C. $PM_{10}$ unreacted, D. $PM_{150}$ SGF 1 h, E. $PM_{150}$ SGF 7 day, F. $PM_{150}$ unreacted, fit components anglesite and plumbojarosite shown. Fits to $\chi(k)\cdot k^2$ EXAFS given in Table S9.

**Table S9.** Lead $L_{III}$ linear combination fits to $\chi(k)\cdot k^2$ EXAFS

| | Pb-Jar[a] | err[b] | Anglesite[c] | err[b] | *R*-factor[d] | red.$\chi^2$ [e] |
|---|---|---|---|---|---|---|
| $PM_{10}$ unrx | 0.863 | 0.028 | 0.137 | 0.013 | 0.04106 | 0.00367 |
| $PM_{10}$ L-7d | 0.858 | 0.029 | 0.142 | 0.016 | 0.06368 | 0.00593 |
| $PM_{10}$ G-1h | 0.765 | 0.030 | 0.235 | 0.020 | 0.12043 | 0.00940 |
| $PM_{150}$ unrx | 0.712 | 0.036 | 0.288 | 0.028 | 0.17302 | 0.00987 |
| $PM_{150}$ L-7d | 0.636 | 0.036 | 0.364 | 0.028 | 0.34196 | 0.01760 |
| $PM_{150}$ G-1h | 0.688 | 0.019 | 0.332 | 0.036 | 0.16880 | 0.00863 |

Fractional fits to reference minerals pre and post IVBA reaction for $PM_{10}$ and $PM_{150}$ in synthetic lung fluid (L) and gastric fluid (G). Samples are unreacted (unrx) and after 7 days (7d) and 1-hour (1h) IVBA. [a] plumbojarosite [$Pb_{0.5}Fe_3(SO_4)_2(OH)_6$] from Root et al. 2015, [b]. estimated error (err) in fit component, [c] anglesite [$PbSO_4$] from Root et al. 2015, [d] *R* factor and [e] reduced.$\chi^2$ chi squared are goodness of fit parameters. Fits shown in Fig. S15**.**

Theoretical phase-shift and amplitude functions were calculated with the program FEFF6 (REHR, 1993) using atomic clutters taken from the crystal structures of ferrihydrite and jarosite for Fe and scorodite and angelellite for As, known Fe and As(V) minerals with geometries similar to those expected for absorber backscatterer interactions of Fe and As in the tailings. Multiple scattering paths (MS) from As (V)–O tetrahedra were included as they have been shown to improve EXAFS fits beyond the first shell for arsenate compounds (BEAULIEU and SAVAGE, 2005; ONA-NGUEMA et al., 2005). During Fe EXAFS fitting, the values of interatomic distance (R, Å) of the Fe-O, Fe-S, and Fe-Fe shells were allowed to vary. The photoelectron threshold energy shift, $\Delta E_0$ (eV), was allowed to float as a common parameter during fit iterations, i.e., a single $\Delta E_0$ parameter was used for all backscatterer paths in a fit. There is a strong correlation between Debye–Waller ($\sigma^2$) and coordination number (N); therefore N was initially held constant during EXAFS fitting based on crystallographic values. Based on empirical fits to known As reference compounds, estimated errors were R ± 0.02 Å, N or $\sigma^2$ ± 30% for atoms beyond the first shell (see O'DAY et al., 2004b). Fe-O was fit to 2-shells; 1.96 A for apical oxygen and 2.08 A for equatorial oxygen and the sum of N for the two oxygen paths was constrained to 6, but distribution and R were allowed to vary in the fit. This allowed the addition of multiple paths without exceeding the Nyquist criterion of independent fit parameters ($N_{idp}$) or increasing the degrees of freedom:

$$N_{inp} = 2\Delta k \Delta R/\pi \qquad \textit{(eq. 1)}$$

**Figure S16**. $PM_{10}$ v bulk tailings elemental concentration

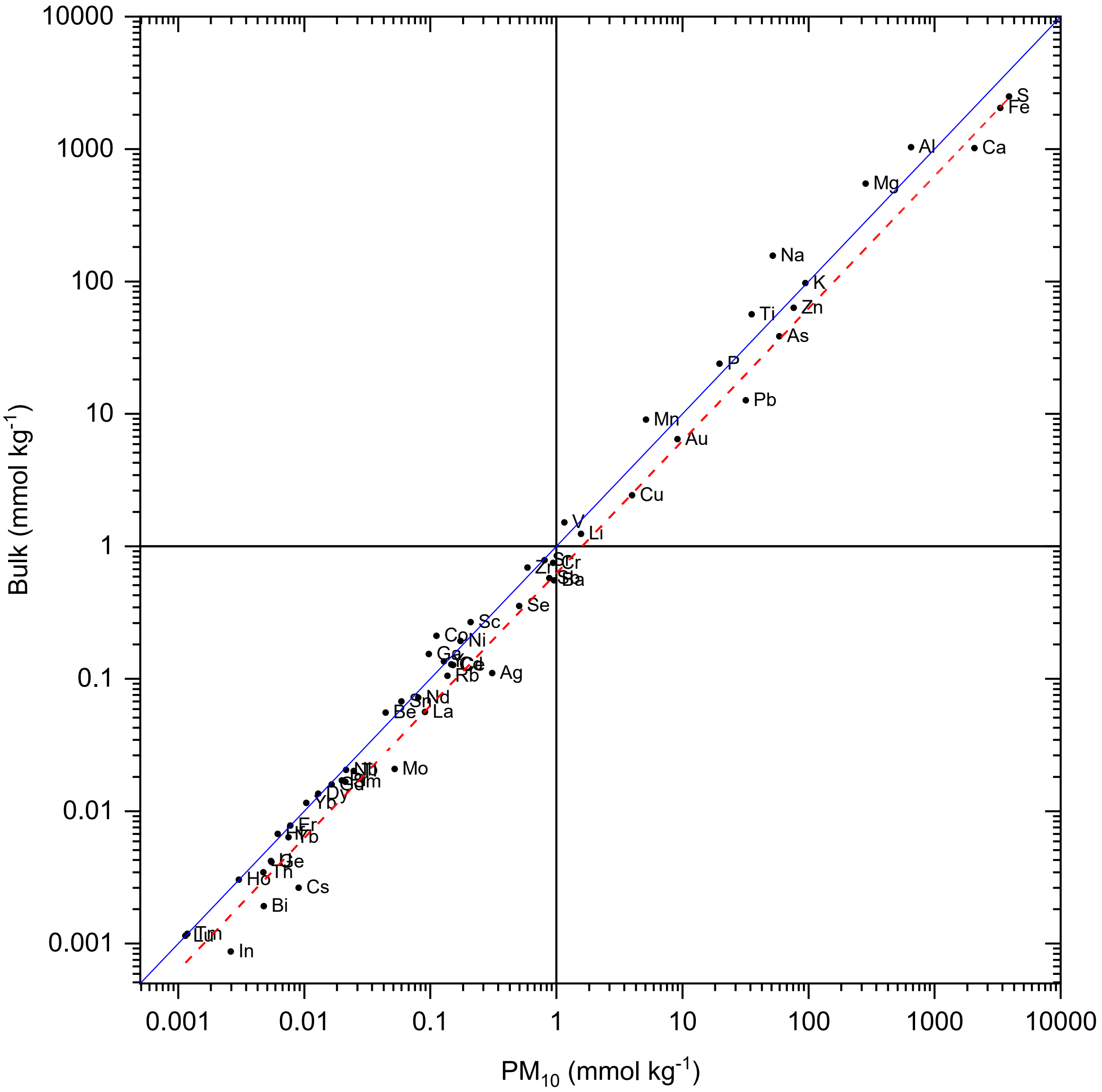


$PM_{10}$ shows an enrichment in elements plotted below the 1:1 blue line. Red dashed line shows linear fit for $PM_{10}$ v bulk tailings for all elements as y = a + bx, a set = 0, b fit = 0.626 ± 0.19, Pearsons's r = 0.9753

**Table S10.** PM$_{10}$ v bulk tailings elemental concentration.

| element | PM$_{10}$ mmol kg$^{-1}$ | Bulk mmol kg$^{-1}$ | PM:bulk ratio |
|---|---|---|---|
| Ag | **0.311** | 0.110 | 2.82 |
| Al | 652 | **1030** | 0.63 |
| As | **58.7** | 38.4 | 1.53 |
| Au | 0.009 | 0.006 | 1.42 |
| Ba | **0.961** | 0.553 | 1.74 |
| Be | 0.044 | 0.055 | 0.80 |
| Bi | **0.005** | 0.002 | 2.50 |
| Ca | **2070** | 1020 | 2.04 |
| Cd | 0.147 | 0.128 | 1.15 |
| Ce | 0.152 | 0.127 | 1.20 |
| Ce | **0.236** | 0.150 | 1.57 |
| Co | 0.112 | **0.210** | 0.53 |
| Cr | 0.942 | 0.750 | 1.26 |
| Cs | **0.009** | 0.003 | 3.43 |
| Cu | **3.99** | 2.42 | 1.65 |
| Dy | 0.013 | 0.014 | 0.95 |
| Er | 0.008 | 0.008 | 1.00 |
| Fe | **3330** | 2040 | 1.63 |
| Ga | 0.098 | **0.153** | 0.64 |
| Gd | 0.017 | 0.016 | 1.04 |
| Ge | 0.006 | 0.004 | 1.33 |
| Hf | 0.006 | 0.007 | 0.92 |
| Ho | 0.003 | 0.003 | 1.00 |
| In | **0.003** | 0.001 | 3.00 |
| K | 94.6 | 97.2 | 0.97 |
| La | 0.072 | 0.056 | 1.28 |
| La | **0.091** | 0.055 | 1.64 |
| Li | 1.57 | 1.24 | 1.27 |
| Lu | 0.001 | 0.001 | 1.00 |
| Mg | 284 | **547** | 0.52 |
| Mn | 5.15 | **9.05** | 0.57 |

| element | PM$_{10}$ mmol kg$^{-1}$ | Bulk mmol kg$^{-1}$ | PM:bulk ratio |
|---|---|---|---|
| Mo | **0.052** | 0.021 | 2.50 |
| Na | 52.2 | **157** | 0.33 |
| Nb | 0.022 | 0.020 | 1.05 |
| Nd | 0.080 | 0.072 | 1.12 |
| Ni | 0.174 | 0.193 | 0.90 |
| P | 19.7 | 23.9 | 0.82 |
| Pb | **31.9** | 12.6 | 2.52 |
| Pr | 0.020 | 0.017 | 1.17 |
| Rb | 0.137 | 0.105 | 1.30 |
| S | **3900** | 2490 | 1.56 |
| Sb | **0.879** | 0.574 | 1.53 |
| Sc | 0.209 | 0.267 | 0.78 |
| Se | 0.508 | 0.355 | 1.43 |
| Sm | 0.018 | 0.016 | 1.13 |
| Sm | 0.021 | 0.017 | 1.28 |
| Sn | 0.059 | 0.067 | 0.88 |
| Sr | 0.808 | 0.785 | 1.03 |
| Ta | 0.001 | <DL | max |
| Te | 0.002 | <DL | max |
| Th | 0.005 | 0.003 | 1.38 |
| Ti | 35.5 | **56.4** | 0.63 |
| Tl | 0.025 | 0.020 | 1.23 |
| Tm | 0.001 | 0.001 | 1.00 |
| U | 0.005 | 0.004 | 1.30 |
| V | 1.16 | 1.51 | 0.77 |
| Y | 0.128 | 0.135 | 0.95 |
| Yb | 0.008 | 0.006 | 1.18 |
| Yb | 0.010 | 0.012 | 0.90 |
| Zn | 76.3 | 63.2 | 1.21 |
| Zr | 0.592 | 0.691 | 0.86 |

**Bold** indicates particle with higher concentration at 1.5 < PM:bulk < 0.75

**References**

O'Day PA, Vlassopoulos D, Root R, Rivera N. The influence of sulfur and iron on dissolved arsenic concentrations in the shallow subsurface under changing redox conditions. Proceedings of the National Academy of Sciences of the United States of America. 2004;101(38):13703-8.

Savage, K.S., D.K. Bird, and P.A. O'Day, Arsenic speciation in synthetic jarosite. Chemical Geology, 2005. 215: p. 473-498.
